# Multimodality imaging of neurodegenerative disorders with a focus on multiparametric magnetic resonance and molecular imaging

**DOI:** 10.1186/s13244-022-01358-6

**Published:** 2023-01-16

**Authors:** James Ryan Loftus, Savita Puri, Steven P. Meyers

**Affiliations:** Department of Imaging Sciences, University of Rochester Medical Center, 601 Elmwood Ave, Rochester, NY 14642 USA

**Keywords:** Neurodegenerative disease, Molecular imaging, Multiparametric MRI, Alzheimer’s disease, Dementia

## Abstract

Neurodegenerative diseases afflict a large number of persons worldwide, with the prevalence and incidence of dementia rapidly increasing. Despite their prevalence, clinical diagnosis of dementia syndromes remains imperfect with limited specificity. Conventional structural-based imaging techniques also lack the accuracy necessary for confident diagnosis. Multiparametric magnetic resonance imaging and molecular imaging provide the promise of improving specificity and sensitivity in the diagnosis of neurodegenerative disease as well as therapeutic monitoring of monoclonal antibody therapy. This educational review will briefly focus on the epidemiology, clinical presentation, and pathologic findings of common and uncommon neurodegenerative diseases. Imaging features of each disease spanning from conventional magnetic resonance sequences to advanced multiparametric methods such as resting-state functional magnetic resonance imaging and arterial spin labeling imaging will be described in detail. Additionally, the review will explore the findings of each diagnosis on molecular imaging including single-photon emission computed tomography and positron emission tomography with a variety of clinically used and experimental radiotracers. The literature and clinical cases provided demonstrate the power of advanced magnetic resonance imaging and molecular techniques in the diagnosis of neurodegenerative diseases and areas of future and ongoing research. With the advent of combined positron emission tomography/magnetic resonance imaging scanners, hybrid protocols utilizing both techniques are an attractive option for improving the evaluation of neurodegenerative diseases.

## Key points

- Neurodegenerative diseases represent a growing substantial burden of disease worldwide.
- Clinical diagnosis and standard imaging techniques lack accuracy in diagnosing dementia syndromes.
- Multiparametric magnetic resonance and molecular imaging are tools for evaluating neurodegenerative disease.
- Combined positron emission tomography/magnetic resonance imaging protocols provide the ideal dementia appraisal.

## Background

Neurodegenerative disease (NDDs) including dementia syndromes represent a substantial burden of disease worldwide. Estimated global prevalence of all-cause dementia is 700 per 100,000 persons with the number of patients with dementia nearly doubling every five years [1]. Clinical diagnosis alone remains not entirely reliable with a median sensitivity of 87% and specificity of 58% [2]. Proper diagnosis is crucial to aid with prognostication and pharmacologic management of patients with NDDs, with disease modifying therapies an active field of research interest and recent US Food and Drug Administration approval of aducanumab, an IgG1 anti-amyloid-beta antibody targeting amyloid beta plaques, the first therapy of its kind available, though not yet approved in Europe [3–6]. Imaging is often applied to increase diagnostic confidence in the setting of a suspected NDD and in 2011 the US National Institute on Aging and the Alzheimer’s Association incorporated imaging biomarkers into the guidelines for diagnosis of Alzheimer’s disease (AD) [7]. Structural imaging methods such as magnetic resonance imaging (MRI) are often undertaken first, though limited sensitivity and specificity and high interobserver variability limit the applicability of these more commonly used methods [8]. Molecular imaging provides promise in its unique ability to visualize the spatial distribution of pathologic changes in NDDs and has been demonstrated to lead to increases in diagnostic certainty and provide therapeutic guidance. We aim to provide a brief review of the characteristics and epidemiology of NDDs, as this topic has been covered in detail elsewhere, and focus on their imaging features across multiple modalities, particularly advanced multiparametric MRI and molecular imaging.

## Alzheimer’s disease

Alzheimer’s disease is the most common form of progressive dementia which accounts for up to 60% of cases in patients older than 65 years [9]. Alzheimer’s disease is pathologically defined by senile gray matter plaques consisting of neurotoxic deposits of extracellular amyloid–beta (Aβ) 42 protein, intracellular neurofibrillary tangles (NFTs) including the three repeat (3R) and four repeat (4R) tau isoforms, and decreased neuronal density from neuronal death. Neurofibrillary changes tend to progress in an orderly manner starting in the transentorhinal cortex and progressing through the medial temporal lobes to neocortical association areas in the frontal, parietal, and occipital lobes [10, 11]. Sporadic and familial forms have been defined with approximately 10% of patients related to presenilin 1 (PSEN1), presenilin 2 (PSEN2), and APOE*E4 genes. The clinical course of Alzheimer’s disease typically begins with a slow decline in memory followed by diminishing function in language, visuospatial, and executive abilities [9, 12]. Patients typically have poor recent memory; disorientation to time and place; impaired recall, recognition, object and space perception, conversational speech, and working memory; and word retrieval difficulty.

**Fig. 1:**
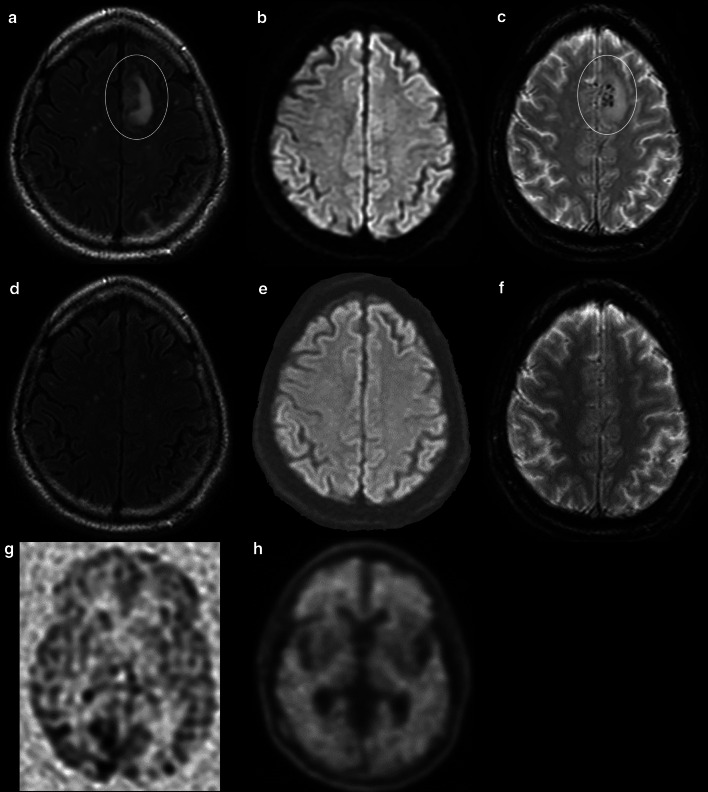
A 67-year-old man with AD on anti-Aβ monoclonal antibody trial. T2 FLAIR image shows hyperintensity in the parasagittal left frontal lobe (oval in **a**), with no evidence of hyperintensity on DWI (**b**), and associated subcortical microhemorrhages (oval in **c**). None of these abnormalities were present prior to receiving antibody therapy (**d**–**f**), consistent with ARIA with edema and hemorrhage. Arterial spin labeling demonstrates hypoperfusion to the parietal and frontal lobes (**g**), with corresponding diffuse cortical uptake on florbetapir PET/CT typical of AD (**h**)

Structural imaging in the evaluation of AD is typically performed with volumetric T1-weighted imaging. Various scales and grading systems have been studied including those evaluating atrophy of the medial temporal lobes [13, 14]. Overall, these have shown adequate sensitivity and specificity, however, are limited by interobserver variation [8]. Automated segmentation methods have also shown the ability to discriminate between AD and controls [15, 16]. Image analysis software with automated volumetric segmentation of brain regions has also shown value for the prediction of development of AD from mild cognitive impairment (MCI), a condition characterized by decline in performance on standardized neurocognitive testing, demonstrating an area under the curve between 0.6 and 0.77 [17, 18]. Structural imaging is also valuable in following patients on monoclonal anti-Aβ antibody therapy, as pooled analysis reported approximately 40% of patients on aducanumab develop amyloid-related imaging abnormalities (ARIAs) (Fig. 1) [19].

Advanced non-molecular imaging techniques have also been researched for the diagnosis of AD. Diffusion tensor imaging (DTI) has shown decreased fractional anisotropy (FA) and increased mean diffusivity (MD) in medial temporal lobe structures including the hippocampus, parahippocampal cingulum, uncinate fasciculus, and fornix as well as the posterior cingulate cortex (PCC), splenium of the corpus callosum, and superior longitudinal fasciculus [20, 21]. Magnetization transfer imaging (MTI), which relies on the exchange of magnetization between protons bound to macromolecules and free water, is another advanced MRI biomarker for evaluating AD, with values often reported as a magnetization transfer ratio (MTR) calculated by subtracting the signal of tissue prior to the pulse sequence by the signal following the pulse sequence then dividing by the signal prior to the pulse sequence [22]. Decreased MTR has been reported in multiple brain regions in AD including the locus coeruleus (LC), hippocampus, entorhinal cortex, precuneus, and global gray matter [22–24]. The proposed mechanism of decreased MTR in the LC is loss of neuromelanin and increased free water, with the LC potentially one of the earliest structures afflicted by NFTs [25, 26]. Decreased N-acetylaspartate (NAA)/myo-inositol ratio in the PCC may predict development of AD [27]. Arterial spin labeling (ASL) has been shown to be comparable to 2-[18F]fluoro-2-deoxy-d-glucose positron emission tomography (FDG-PET) in the diagnosis of AD, however, with lesser diagnostic performance in MCI [28]. Resting-state functional MRI (rsfMRI) has fairly consistently shown hypoconnectivity in the default mode network (DMN), which can be seen in earlier stages of the disease process, especially in impacted mutation carriers [29]. Decreased stiffness within the temporal and parietal lobes on magnetic resonance elastography (MRE) has been reported [30].

**Fig. 2:**
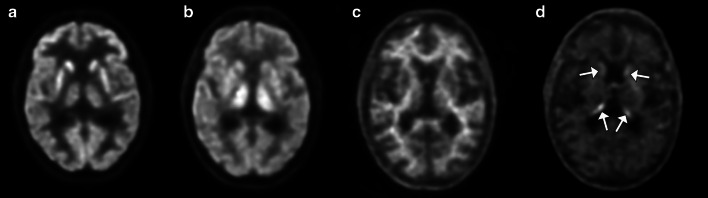
Normal patterns of cerebral uptake across multiple radiotracers. Fluorodeoxyglucose PET/CT demonstrates high-grade uptake in the cortical and deep gray matter which should be greater than cerebellum (**a**). Cerebral blood flow imaging with early-frame or dynamic amyloid agents shows a similar pattern to FDG (**b**). Non-specific low-grade white matter activity with absence of gray matter activity is the normal pattern of delayed amyloid PET agents (**c**). Normal distribution of tau agents is low-grade gray and white matter activity with absence of elevated neocortical uptake and a variable amount of off-target binding, commonly involving the basal ganglia and choroid plexuses (arrows in **d**)

Recently, molecular imaging has shown promise in improving diagnostic confidence in AD. Current targets of clinically used radiotracers include glucose metabolism, amyloid beta plaques, and tau. Figure 2 demonstrates normal patterns of radiotracer activity for these agents. Patient preparation, dosing and kinetics, and normal and abnormal distribution of the commonly used radiotracers are described in Table 1.

**Table 1:** Commonly used radiotracers in evaluation of neurodegenerative syndromes

	FDG-PET	Amyloid PET	Tau PET
Patient Preparation	4 to 6 h fasting Abstain from heavy exercise for 24 h prior to injection Following injection sit quietly in dimly lit room with eyes open	No special preparation	No special preparation
Administered activity MBq (mCi)	185–740 (5–20)	F-18-Florbetapir 370 (10) F-18-Florbetaben 300 (8) F-18-Flutemetamol 185 (5)	F-18-Flortaucipir 370 (10)
Effective dose	0.019 mSv/MBq	~ 6–7 mSv	~ 8–9 mSv
Uptake period (minutes)	30–60	F-18-Florbetapir 30–50 F-18-Florbetaben 45–130 F-18-Flutemetamol 90 *Note dynamic imaging immediately following bolus can also be performed to estimate cerebral blood flow	F-18-Flortaucipir 80
Acquisition time (minutes)		F-18-Florbetapir 10 F-18-Florbetaben 15–20 F-18-Flutemetamol 20	F-18-Flortaucipir 20
Normal study	Uptake within the cerebral cortex and deep gray nuclei greater than cerebellum	Uptake within the cerebral white matter	Absence of neocortical uptake. Note there can be significant off-target binding in the striatum, choroid plexus, and brainstem nuclei which is considered normal
Abnormal study	Regions within the cerebral cortex and deep gray nuclei with decreased metabolic activity compared to cerebellum (with the exception of Parkinson plus syndromes which lead to cerebellar hypometabolism)	Uptake in the cerebral white matter with blurring of the corticomedullary junction, with various named signs (tree in summer, kissing hemispheres, etc.)	Increased contiguous neocortical activity, generally involving greater than one area

*PET* Positron emission tomography, *MBq* Megabecquerel, *mCi* Millicurie, *mSv* Millisievert

**Fig. 3:**
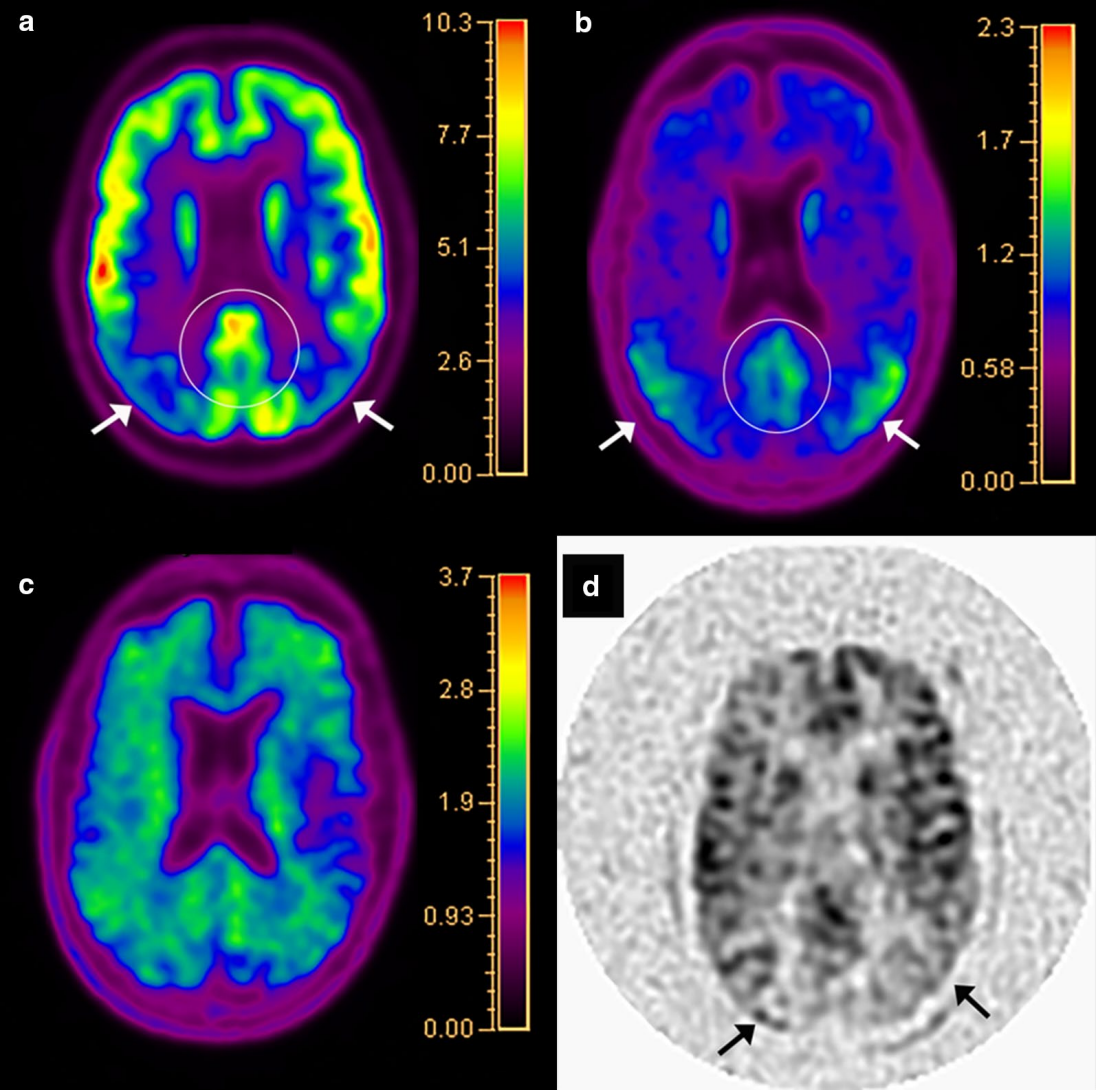
A 73-year-old man with AD, Mini Mental State Examination (MMSE) 23/30. Fluorodeoxyglucose PET/CT (**a**) demonstrates hypometabolism involving the bilateral parietal lobes (arrows) and precuneus and PCC (oval). Flortaucipir PET/CT (**b**) shows tau retention in the bilateral parietal lobes (arrows) and precuneus and PCC (oval), mirroring the regions of hypometabolism. Florbetapir PET/CT (**c**) demonstrates diffuse cortical uptake with “kissing hemispheres.” Arterial spin labeling (**d**) demonstrates hypoperfusion to the bilateral parietal lobes (arrows), similar to the FDG-PET/CT

**Fig. 4:**
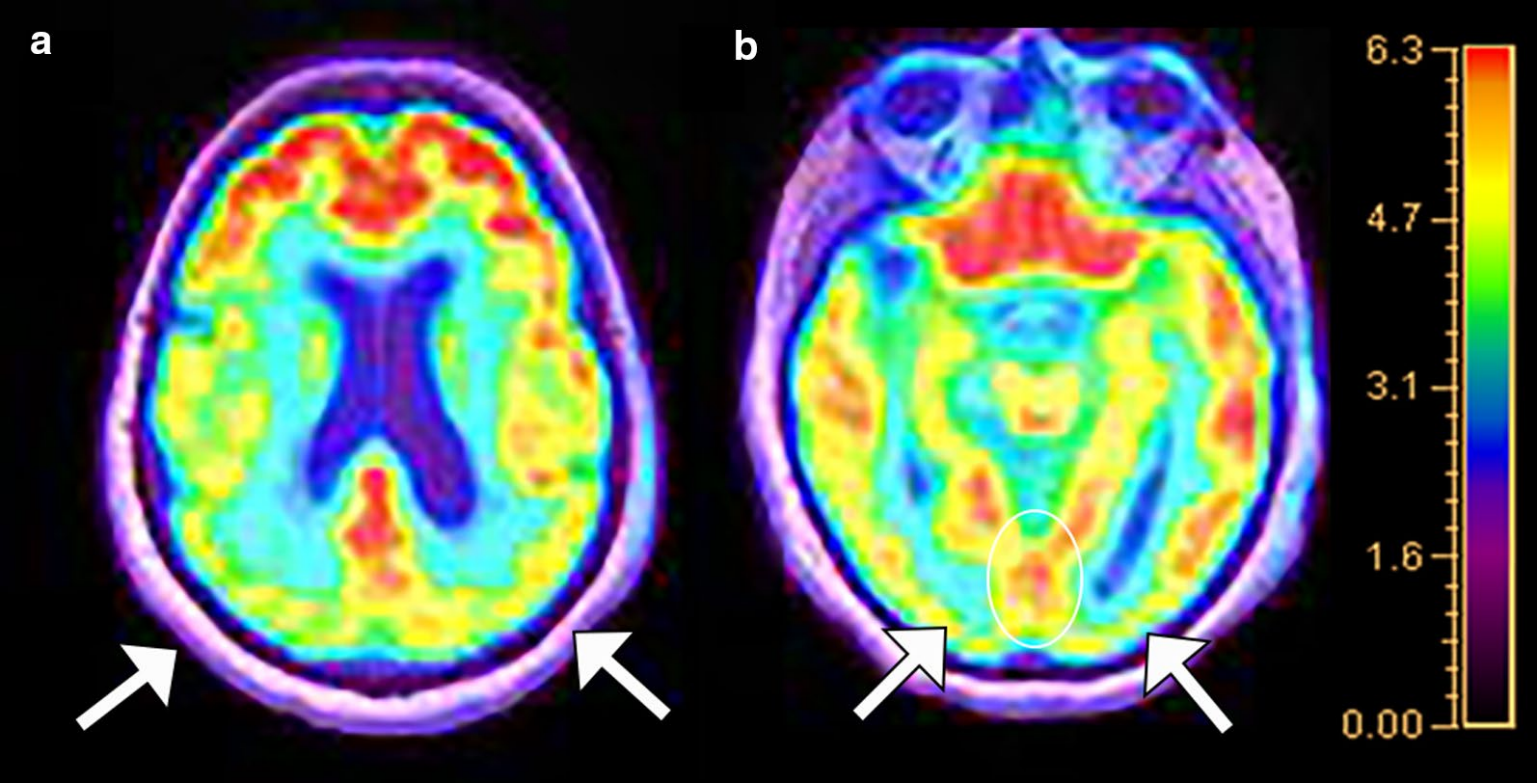
A 76-year-old woman with history of memory loss. Fluorodeoxyglucose PET/MRI (**a**) demonstrates hypometabolism of the parietal lobes (arrows), precuneus, and PCC as well as the visual association centers in the lateral occipital lobes (arrows in **b**). Metabolic activity is preserved in the medial occipital lobes (“occipital tunnel sign,” white oval in **b**). Pattern of hypometabolism is most suggestive of posterior cortical atrophy

Fluorodeoxyglucose PET imaging has been used as a discriminatory tool for the diagnosis of AD since the early 2000s. Hypometabolism on FDG-PET scans has shown to be a reliable indicator of neuronal degeneration in AD [7]. A meta-analysis demonstrated excellent performance of FDG-PET in discriminating between AD and non-AD neurodegenerative syndromes with a sensitivity of 90% and specificity of 89% [31]. Additionally, FDG-PET has also shown signs of value in predicting the progression from amnestic type MCI to AD, especially when single-subject statistical parametric mapping (ss-SPM) was utilized, with a low probability of progression in three years with a negative study [32, 33]. Typical patterns of hypometabolism in MCI and early typical AD include the temporal lobes, parahippocampal gyri, PCC, and precuneus, with involvement of the precuneus and middle and inferior temporal gyri more characteristic of AD [34–36]. Advanced typical AD shows a fairly consistent pattern of diffuse hypometabolism involving the aforementioned areas as well as the parietal lobes and prefrontal cortex with relative sparing of the precentral gyrus, basal ganglia, and occipital cortex (Fig. 3), except in the posterior cortical atrophy (PCA, also known as Benson’s) clinical phenotype of Alzheimer’s dementia, where lateral occipital lobe atrophy and hypometabolism creates the “occipital tunnel” sign (Fig. 4) [37, 38]. Limbic-predominant AD tends to have more pronounced hypometabolism in the hippocampus and related mesial temporal lobe structures with additional involvement of the frontal cortex, while the limbic-sparing or cortical-predominant subtype involves similar regions to typical AD, with more prominent involvement of the frontal lobes and lesser involvement of the mesial temporal lobes as its name suggests [39]. Additional clinical AD phenotypes which can be discriminated on FDG-PET include the logopenic variant of primary progressive aphasia (lvPPA) which shows left/dominant hemisphere posterior perisylvian or parietal hypometabolism and the dysexecutive/behavioral variant which has similar temporoparietal hypometabolism to typical AD however with variable involvement of the PCC and frontal lobes [40, 41].

Along with qualitative assessment, semiquantitative assessment of FDG-PET can be performed by calculating the cerebral metabolic rate of glucose (CMRgluc), formally calculated by diving the plasma glucose level by a “lumped constant,” to correct for the varying affinities of FDG and glucose for the hexokinase transporter, multiplied by the rate of transfer of FDG from blood to brain [42]. As the method is invasive and requires numerous blood draws, other methods of calculation have been developed to approximate CMRgluc and standard uptake value of glucose (SUVgluc) [43, 44]. Studies have linked the degree of decreased glucose metabolism with the severity of cognitive impairment on standardized mental status examinations [45, 46].

Single-subject statistical parametric mapping can also be applied to the FDG-PET data set to better quantitate the degree of abnormality of an individual scan. This technique allows for voxel-based analysis through creation of a statistical map of an individual’s scan and comparing the uptake values on that scan to a series of control scans [47]. Multiple groups have documented increased diagnostic performance with ss-SPM of FDG-PET/CT [48, 49].

**Fig. 5:**
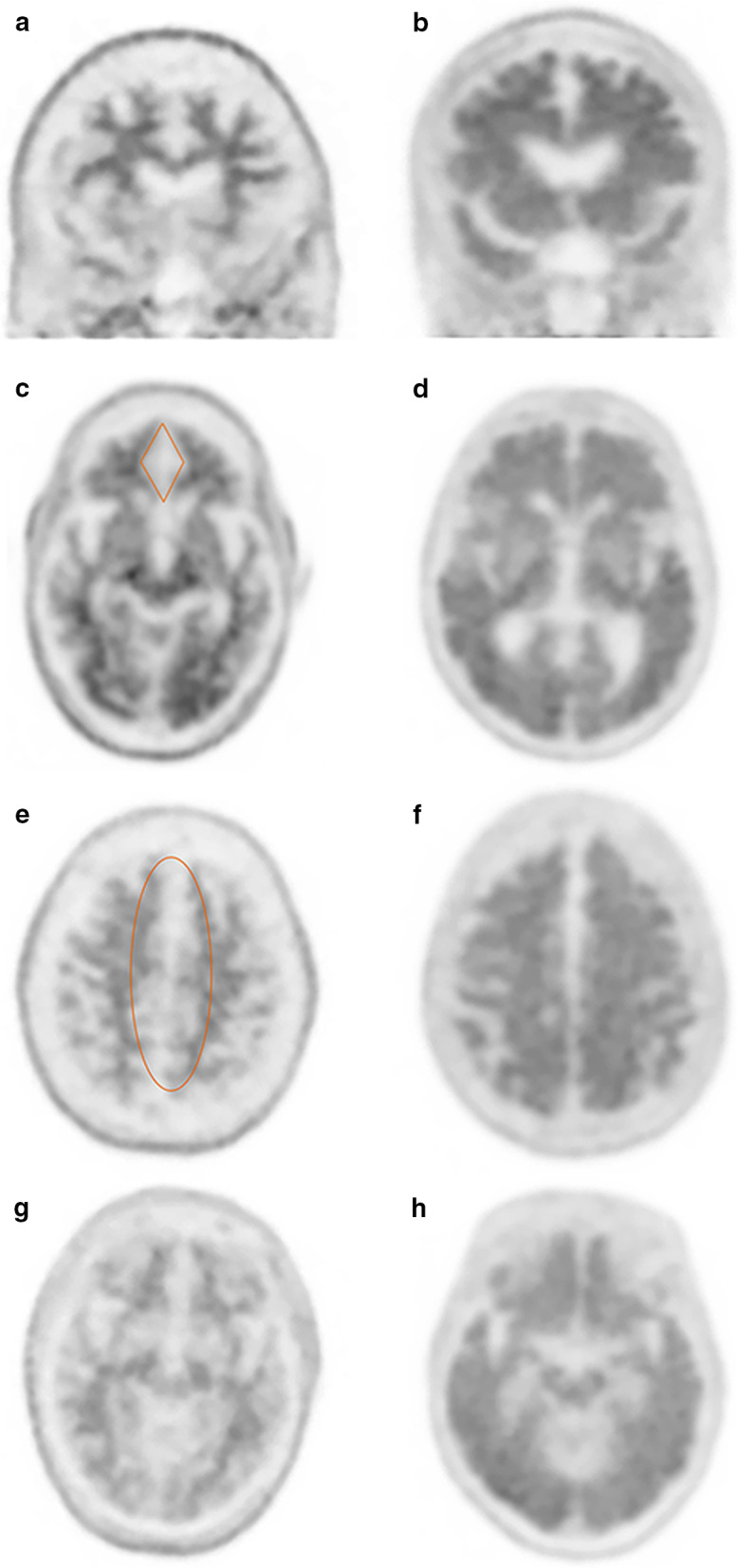
Named signs of normal and abnormal patterns of amyloid activity on florbetapir PET/CT. Normal activity in the white matter of the frontal lobes leads to the “tree-in-winter sign” on coronal images, abnormal activity in the gray matter leads to the appearance of leaves known as the “tree-in-summer sign” (**a**, **b**). Absence of gray matter activity in the medial orbitofrontal lobes creates a diamond pattern of the white matter activity which is lost in abnormal studies (**c**, **d**). A similar phenomenon is seen at the cerebral convexities where the superior white matter tracts create the “double convex lens sign” (**e**) which is lost with abnormal gray matter activity morphing to the “kissing hemispheres sign” (**f**). Lastly, normal absence of gray matter activity in the temporal and occipital lobes creates a mountainous façade referred to as the “temporo-occipital ridge” (**g**), whereas abnormal gray matter activity leads to a more flat profile termed the “temporo-occipital plain” (**h**)

**Fig. 6:**
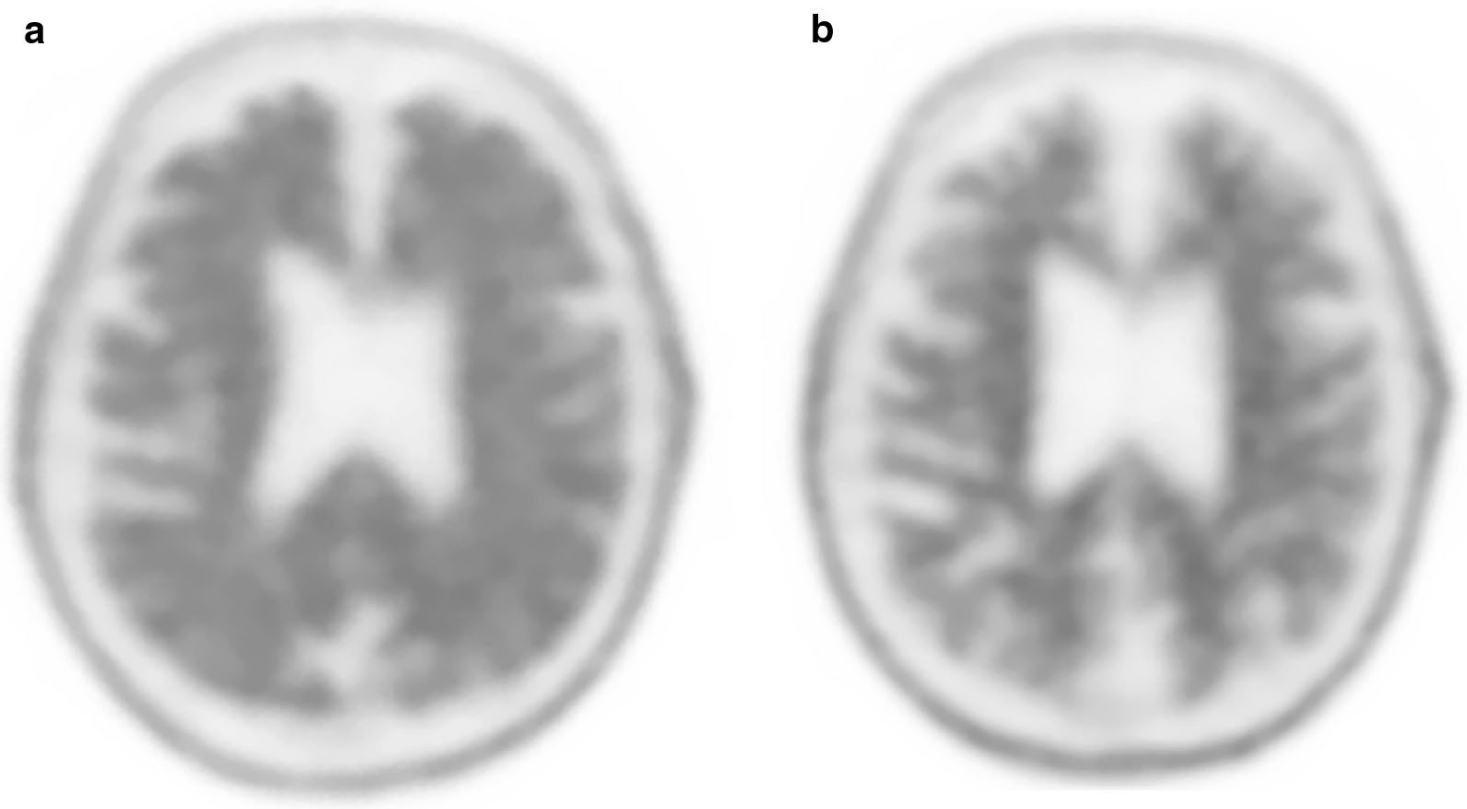
A 83-year-old male with typical AD on anti-Aβ monoclonal antibody clinical trial drug. Pre-treatment florbetapir PET/CT demonstrates substantial cortical amyloid burden with kissing hemispheres in midline (**a**). Following treatment there is significant reduction in amyloid uptake with visualization of corticomedullary differentiation reminiscent of a normal scan (**b**)

In comparison to FDG, Aβ selective radiotracers provide a disease-specific means of imaging the pathologic changes of AD in vivo. Carbon-11 (C-11) Pittsburgh compound B (PiB) was the first amyloid radiotracer used in human studies; however, the short half-life of C-11 limited its clinical applicability and fluorine-18 (F-18) labeled tracers were later produced [50]. Florbetaben (Neuraceq, Piramal, Mumbai), florbetapir (Amyvid, Eli Lilly, USA), and flutemetamol (Vizamyl GE Healthcare, USA) are commonly used F-18 labeled Aβ selective radiotracers, with third-generation tracers in development and preclinical trials. These tracers have also shown robust ability to distinguish AD from controls with pooled analysis demonstrating a sensitivity and specificity of 90% and 87% for florbetapir and 89% and 88% for florbetaben [51]. Amyloid imaging has shown added value to FDG-PET and clinical evaluation [52]. Qualitative interpretation of amyloid imaging is either considered positive (moderate to frequent amyloid plaque) or negative (no to sparse amyloid plaque) based on abnormal gray matter uptake on grayscale imaging in one or two regions for florbetaben and florbetapir respectively, or one region on rainbow color scale for flutemetamol [53–55]. Normal studies exhibit physiologic uptake within the white matter with the absence of uptake in the gray matter creating many named imaging signs, including the “diamond” of the white matter tracts of the orbitofrontal gyri, “cartoon hand” and “tree in winter” in the white matter of the frontal lobes, “double convex lens” involving the frontal and parietal parasagittal region, and the “temporo-occipital ridge” (Fig. 5) [56]. Abnormal regions demonstrate radiotracer activity within the gray matter, leading to blurring of the gray matter-white matter junction with most common sites including the precuneus, PCC, and lateral temporal and parietal lobes. The activity within the gray matter leads to the appearance of “kissing hemispheres” along the interhemispheric fissure, “tree in summer” when viewed in the coronal plane, and a “temporal plain” as opposed to a “temporo-occipital ridge” (Fig. 5) [9, 12, 56]. Even small amounts of amyloid plaques may be predictive of eventual development of AD [57]. Positron emission tomography amyloid imaging has shown similar diagnostic accuracy to cerebrospinal fluid (CSF) analysis, with the obvious advantage of negating an invasive procedure [58]. Amyloid imaging may also be used to follow patients on monoclonal antibody therapy to demonstrate clearance of Aβ plaques (Fig. 6).

Semiquantitative analysis can also be performed for PET amyloid studies by calculating a standardized uptake value ratio (SUVr). This is achieved by first selecting a region of interest to determine baseline, most often the cerebellar gray matter as this rarely accumulates amyloid, and comparing the ratio of uptake in this region to specified regions of interest (ROIs) [59]. If the region demonstrates a SUVr above a predetermined threshold, it is considered positive for moderate to frequent amyloid plaque [60]. Currently there is debate in the literature about whether qualitative or semiquantitative interpretation of amyloid imaging is more efficacious [61, 62]. Additionally, as with FDG-PET, ss-SPM can be undertaken to increase diagnostic confidence. While the literature at the time is sparse, coupling these techniques with amyloid PET imaging has shown promising results in terms of diagnostic accuracy, with sensitivity and specificity near or surpassing 90% [63]. Dynamic imaging can also be performed immediately following the bolus to estimate cerebral blood flow, with a report suggesting decreased early time frame amyloid activity correlating with tau retention [64].

**Fig. 7:**
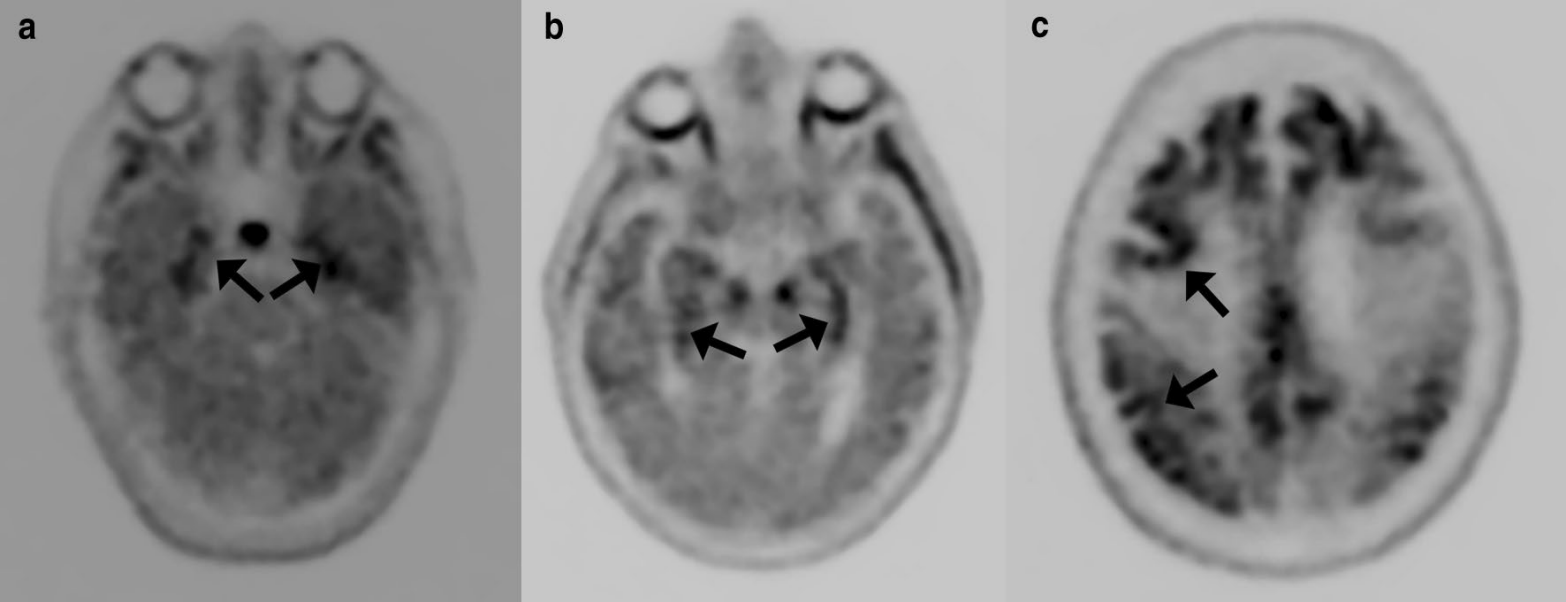
In vivo Braak staging on flortaucipir PET/CT. Activity confined to the entorhinal cortices and hippocampi falls into the Braak I-II stages (arrows in **a**), further progression in the temporal lobes and limbic cortices classifies stages III-IV (parahippocampal gyri; arrows in **b**), and neocortical activity defines stages V-VI (arrows in **c**)

Tau selective radiotracers can also be utilized for disease-specific imaging of AD. Currently, the most widely used tau selective radiotracer is older-generation F-18 flortaucipir (Tauvid; Avid Radiopharmaceuticals, previously known as AV 1451 and T807) [65]. The limitation of this older-generation tau radiotracer is substantial off-target binding, with one study reporting 64% of the signal in amyloid negative healthy controls was due to off-target binding primarily from the basal ganglia structures and choroid plexuses [66]. Off-target binding can also occur in the muscles and secondary to monoamine oxidase (MAO) enzymatic activity in astrocytes in non-specified neuroinflammation. Newer-generation tau radiotracers including F-18RO-948 (previously RO69558948), F-18-MK6240, F-18-PI2620, and F-18-GTP1 are under investigation [67–71]. Carbon-11 labeled tau radiotracers are also being studied, although as with PiB, C-11 labeled radiotracers will inherently be limited by short half-life [68, 72]. Fluorine-18-RO-948, F-18-MK6240, and F-18-GTP1 have been shown to have greater affinity for tau than F-18 flortaucipir [70, 72, 73]. In contrast to amyloid imaging, tau imaging allows for better visualization of the topography of the pathologic changes of AD. The earliest regions of radiotracer uptake are in the mesial temporal lobes including the entorhinal cortices and hippocampi, progressing to the middle and inferior temporal lobes, parietal lobes including the angular and supramarginal gyrus, cingulate cortex, and dorsolateral frontal lobes, with groups showing promise of in vivo Braak staging using tau PET imaging [74, 75]. A recent study demonstrated a sensitivity of 92.3% to 100.0% and specificity ranging from 52.0 to 92.0% for identifying Braak stage V or VI disease at postmortem evaluation for F-18 flortaucipir (Fig. 7) [76]. Tau imaging may also differentiate between AD subtypes better than amyloid and closely mirrors cortical gray matter atrophy and FDG-PET hypometabolism [77–79]. As with amyloid radiotracers SUVr values can be calculated, again generally using the cerebellum for the reference value [80].

Lastly, ongoing research is being performed to utilize molecular imaging for the evaluation of neuroinflammation and synaptic density in AD with a variety of C-11 and F-18 labeled radiotracers, none of which are currently widely used clinically. An 18 kDa translocator protein is the typical target for microglial activation with the most widely used tracer 11C-PK11195 [81]. Astrocytosis in AD is imaged by targeting MAO-B with 11C-deuterium-l-deprenyl (11C-DED) [82]. Studies have reported increased and no significant difference in microglial activation in AD patients when compared to controls [83–86]. Temporal changes in astrocytosis have been reported, with increased radiotracer activity in prodromal AD followed by a gradual decline in activity as the disease progresses which correlates with hypometabolism [87–89]. Uptake of radiotracers 11C-UCB-J and 18F-UCB-H, which bind to the synaptic glycoprotein 2, have been shown to be reduced in the hippocampi of patients with AD [90–93].

## Dementia with Lewy bodies

Dementia with Lewy bodies (DLB) is considered the second or third most common dementia, accounting for approximately 5% of all dementia cases in incidence studies, and up to approximately 20% of all dementia cases in prevalence studies [94]. Clinically DLB is defined by fluctuating cognition, visual hallucinations, rapid eye motion sleep behavior disorder, and parkinsonism [95]. Pathologically DLB is defined by loss of dopaminergic neurons in the substantia nigra with reduced striatal dopaminergic activity and neuronal inclusions of alpha-synuclein-positive Lewy bodies in the cerebral cortex, substantia nigra, and brainstem. These findings are indistinguishable from Parkinson’s disease (PD); however, the involvement of the cortex may be more pronounced in DLB and neuronal loss in the substantia nigra more prominent in PD [96]. In addition, there are often concomitant Aβ plaques and 3R/4R NFTs and over-expression of the APOE*E4 genotype, overlapping with the pathologic features of AD [97, 98].

As with AD, structural MRI plays a role in the assessment of DLB. T1 volumetric analysis of patients with DLB demonstrates atrophy of the frontal and temporal lobes and insular cortices with less pronounced involvement of the medial temporal lobe when compared to AD, hypothesized to be related to comparatively reduced NFT formation [99, 100]. Sparse partial least squares classification of cortical thickness based on T1-weighted imaging demonstrated good discrimination of DLB from AD with a sensitivity of 78% and specificity of 75% [101]. Atrophy of the midbrain, hypothalamus, and substantia innominate have also been shown to be useful in discriminating DLB from AD [102].

**Fig. 8:**
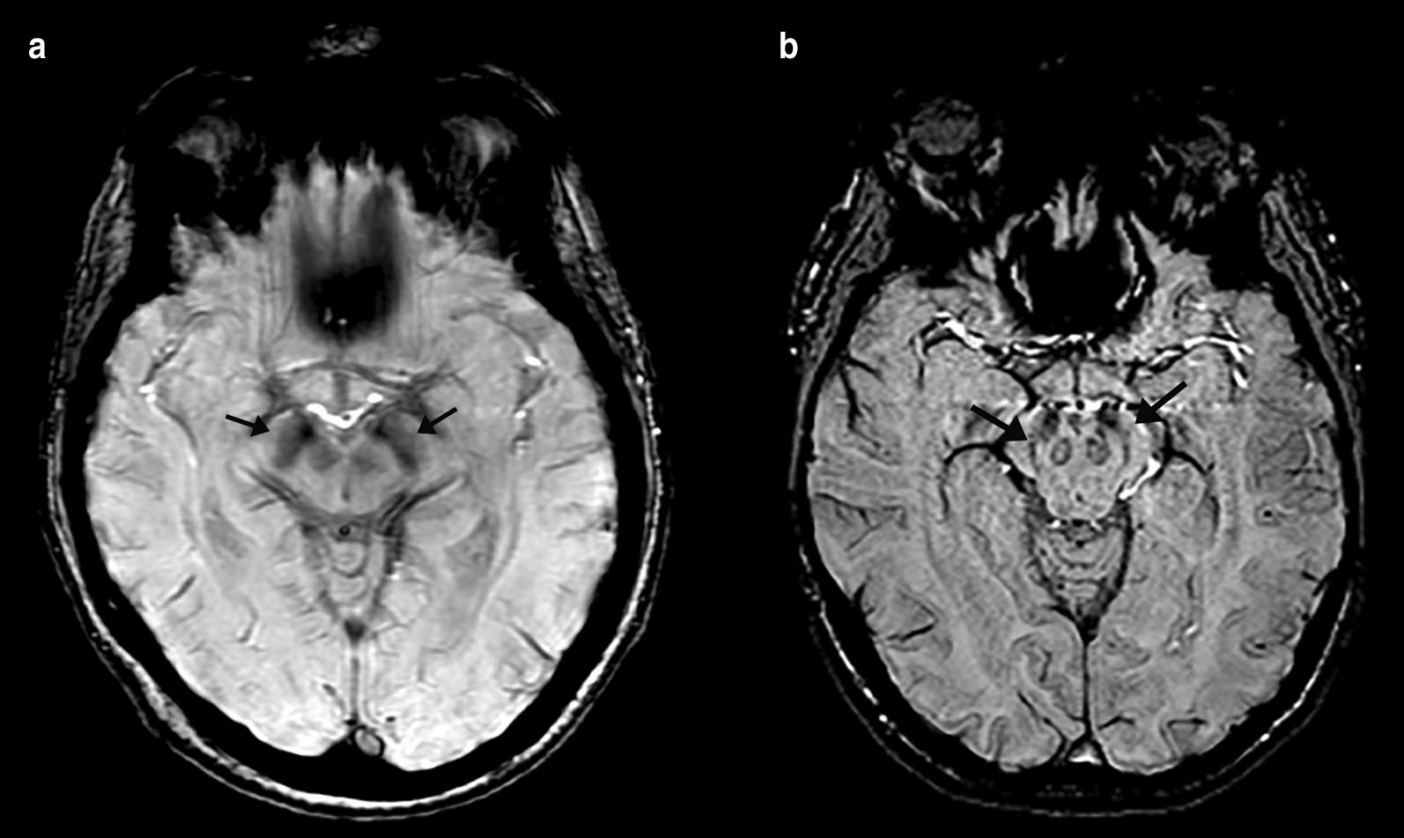
A 82-year-old female with history of rigidity, bradykinesia, hallucinations, and dementia. Axial susceptibility-weighted imaging demonstrates loss of the normal hyperintense signal in nigrosome-1 (arrows), the so-called loss of “swallow tail sign” (**a**). The normal hyperintense signal in nigrosome-1 is shown in (**b**) (arrows)

Advanced MRI techniques have also been reported to be of value in the diagnosis of DLB. Studies have shown decreased FA involving the cortical and subcortical regions including the parieto-occipital lobes, PCC, precuneus, inferior longitudinal fasciculus, caudate, putamen, and pons [103–106]. Abnormalities in FA in the parietal and occipital regions in DLB have been shown to not significantly change over time when compared to controls, corroborating the theory DLB is primarily associated with synaptic dysfunction rather than neuronal loss [107]. Absence of the “swallow tail sign,” hypointensity within nigrosome-1 on susceptibility-weighted imaging, has been reported to have a sensitivity of 63–93% and a specificity of 79–87% in discrimination DLB from other forms of dementia (Fig. 8) [108, 109]. A similar finding was demonstrated with postmortem MTI with lower signal in the substantia nigra pars compacta in patients with DLB and PD, potentially secondary to neuromelanin loss [110]. Regions of decreased contrast-to-noise ratio in the LC on MTI have been shown to relate to symptoms in PD [111]. Similar to AD, rsfMRI exhibits decreased connectivity in the default mode and executive networks and additionally the visual networks including the medial occipital network [112, 113].

Molecular imaging also plays a crucial role in the diagnosis and potentially response to therapy of DLB. Dopamine transporter (DAT) imaging has been a target for DLB, specifically the imaging of the density of the presynaptic striatal neurons to assess for degeneration in presynaptic parkinsonian syndromes. Single-photon emission computed tomography (SPECT) imaging with iodine-123 N-ω-fluoropropyl-2β-carbomethoxy-3β-[4-iodophenyl] nortropane (I-123-FP-CIT, DaTSCAN™, GE Healthcare), a cocaine analogue with high affinity for the dopamine and serotonin transporters allowing for in vivo evaluation of presynaptic striatal neuronal degeneration, was initially used clinically for discriminating PD from essential tremor on the basis of excellent reported specificity [114]. As PD and DLB share the same pathologic basis, I-123-FP-CIT has shown similar value in the diagnosis of DLB, with a diagnostic accuracy around 90% in discriminating from other dementia syndromes; however, DAT imaging is not reliable for discriminating DLB from PD-related MCI or dementia [115, 116]. Single-photon emission computed tomography imaging with I-123-Metaiodobenzylguanidine to assess for cardiac postganglionic sympathetic denervation observed in DLB is reported to have similar sensitivity and specificity to I-123-FP-CIT SPECT, although pre-existing cardiac disease and diabetes mellitus can lead to false positives [117, 118].

**Fig. 9:**
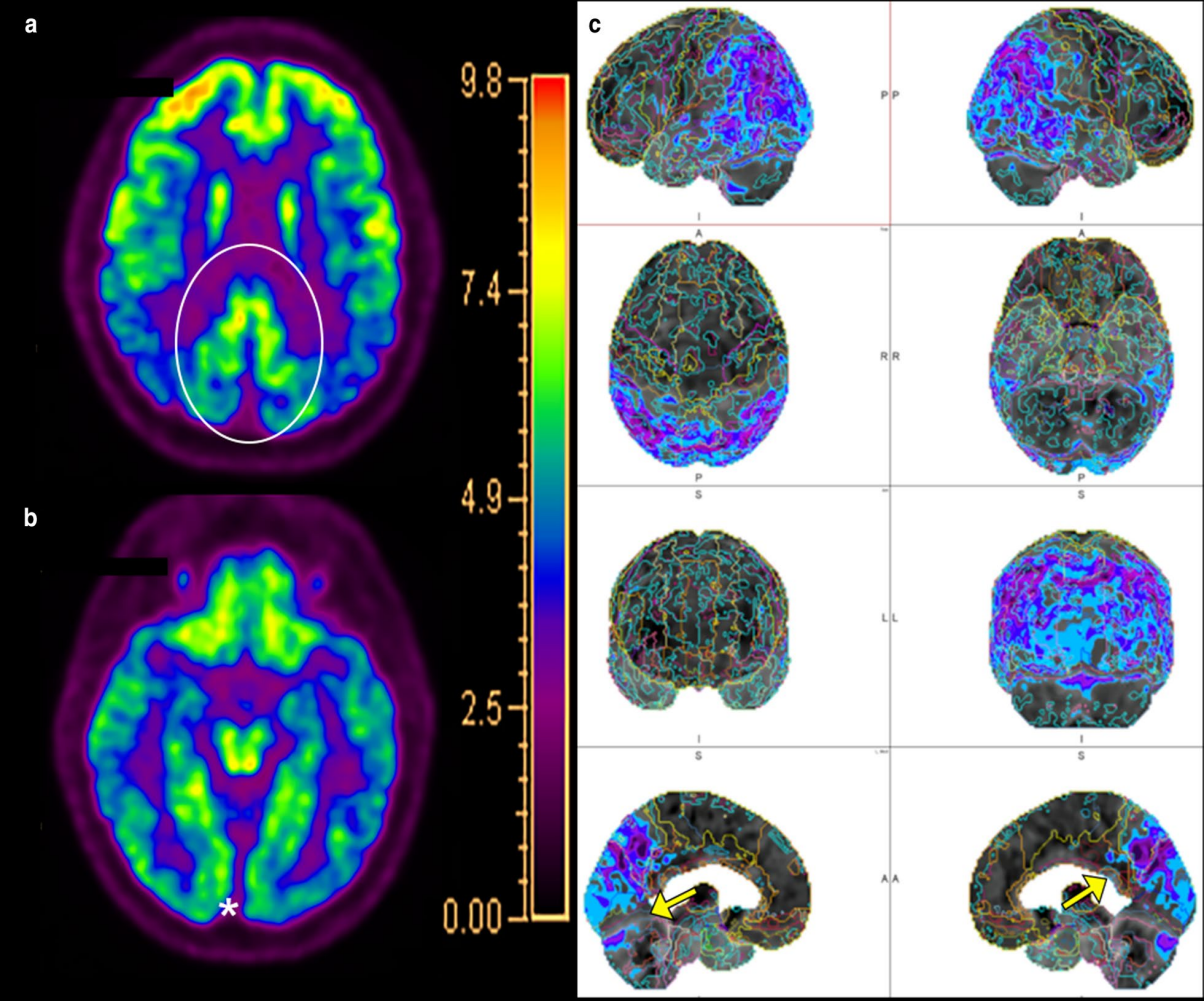
A 73-year-old man with memory loss and visuospatial processing deficits. Fluorodeoxyglucose PET/CT demonstrates hypometabolism in the bilateral parietal lobes including the bilateral precuneus with preserved metabolism of the PCC (cingulate island sign, oval in **a**). Also present is hypometabolism of the bilateral lateral occipital lobes with preserved medial occipital lobe activity (occipital tunnel sign, asterisk in **b**). Single-subject statistical parametric mapping (**c**) demonstrates parietal-occipital hypometabolism reiterating the occipital tunnel (arrow bottom left panel) and posterior cingulate island (arrow bottom right panel), with areas of blue progressing to purple representing further negative deviation from the mean uptake values of controls

Positron emission tomography imaging can also be performed to evaluate for DLB. Decreased metabolic activity in the occipital, temporoparietal, and prefrontal cortices with relative sparing of the pre- and post-central gyri and medial temporal lobes has been reported in DLB [119]. Additionally, there is relatively preserved FDG uptake within the PCC, with surrounding hypometabolic activity in the cuneus and precuneus and parietal lobes, creating the so-called cingulate island sign, reported to have excellent specificity of up to 100% in differentiating AD from DLB when present (Fig. 9) [120]. The “occipital tunnel sign” is also seen in DLB, which is characterized by hypometabolism in the visual association cortex of the lateral occipital lobes and preserved metabolism in the medial occipital lobes [38]. Other studies have demonstrated less promising results for FDG-PET in differentiating DLB from AD, indicating the value of a multimodality assessment [115].

As DLB can pathologically demonstrate Aβ plaques and NFTs, both amyloid and tau tracers can be of value in the imaging of DLB. The majority of DLB patients show amyloid uptake, with a similar pattern to AD involving the dorsolateral frontal lobes, parietal lobes including the precuneus, and temporal lobes, with greater amyloid deposition in the occipital lobes in DLB [121, 122]. Multiple groups have reported the degree of amyloid burden may be related to cognitive decline in DLB and helpful in differentiating DLB from PD-related dementia (PDD) [122–124]. Combined amyloid and dopamine terminal PET imaging was reported to have high accuracy in diagnosing dementia subtype when compared to pathologic examination [125]. Tau PET imaging is less well studied in DLB, with typical areas of increased uptake involving the inferolateral temporal lobes, parietal lobes including the precuneus, and occipital lobes, with decreased degree of uptake and involvement of the medial temporal lobes when compared to AD, with DLB again showing greater abnormality compared to PDD [126, 127]. Attempts at developing a radiotracer to target alpha-synuclein, the pathologic hallmark of DLB, have not been successful at the current time [128–130].

## Vascular dementia

Vascular dementia (VaD) is considered the second or third most common dementia, varying with DLB by source [131]. There are multiple subtypes of vascular dementias, attributed to small vessel and large vessel etiologies as well as acquired and inherited conditions, such as cerebral autosomal dominant arteriopathy with subcortical infarcts and leukoencephalopathy (CADASIL). Clinical presentation is heterogeneous, with the most prominent feature decline in frontal lobe tasks including executive function and attention with verbal memory less impacted [132]. VaD also has a strong correlation with neuropsychiatric symptoms including depression [133].

Structural MRI has long held a central role in the evaluation of VaD. Two or more large territory, three or greater lacunar infarcts, or strategically placed infarcts have been considered sufficient imaging evidence for VaD by various guidelines [134, 135]. Involvement of greater than 25% of a cerebral hemisphere with white matter abnormalities/hyperintensities (WMHs) can be suggestive of subcortical VaD, although there is interobserver variation in making the diagnosis [136, 137]. Dilated Virchow-Robins spaces have also been correlated with the degree of severity of subcortical VaD [138]. Diffusion tensor imaging may predict the structural changes in VaD even prior to development of WMHs and better predicts decline in cognition with reduced FA and increased MD reported in the centrum semiovale and anterior periventricular white matter [139, 140]. Reduced FA and increased MD in the inferior fronto-occipital fascicles, forceps minor, genu, and splenium of the corpus callosum, and the superior longitudinal fasciculus have shown value in discriminating VaD from AD [141]. Early studies of rsfMRI demonstrate dysfunction involving the DMN [142].

**Fig. 10:**
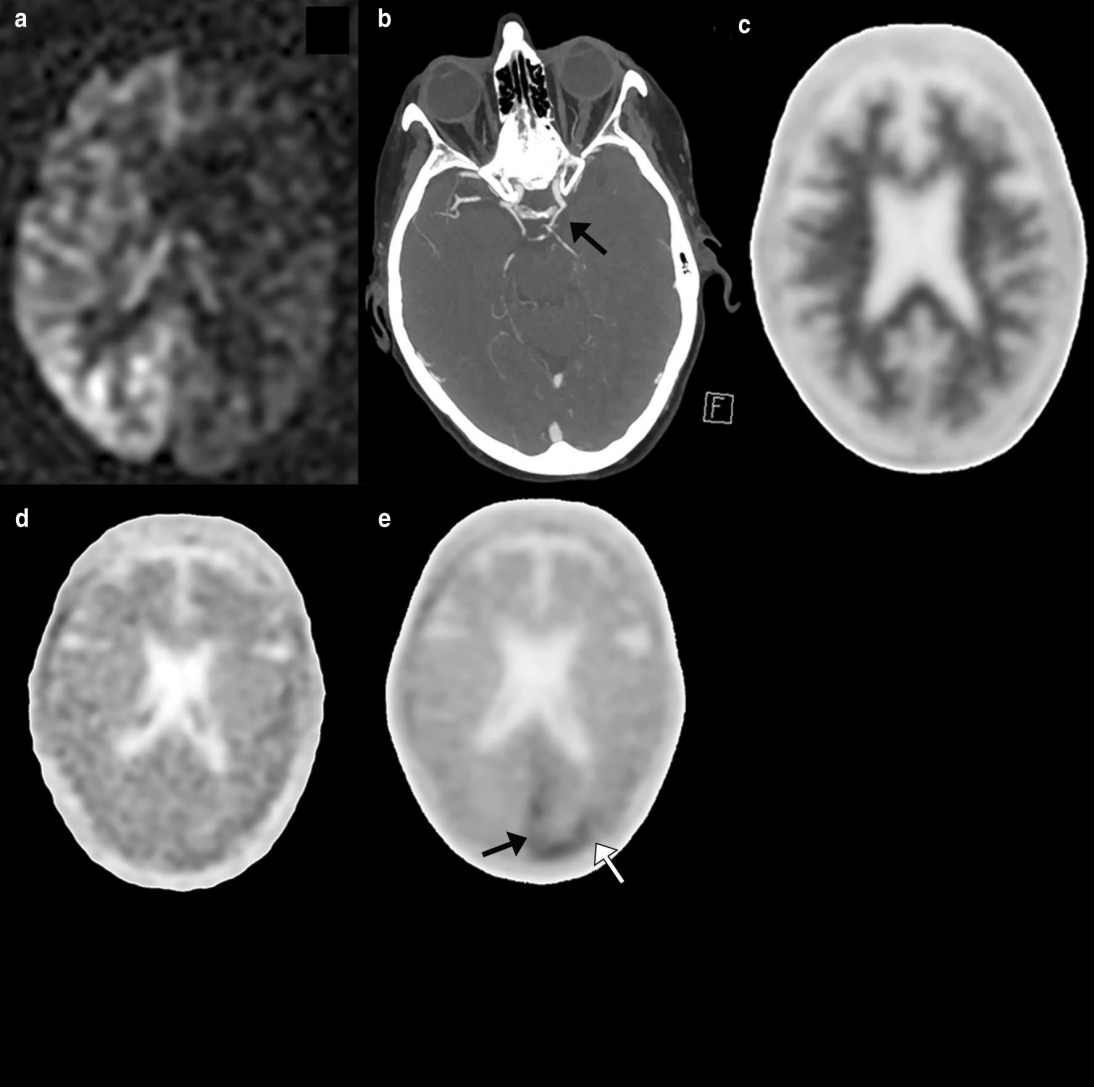
A 76-year-old female with symptomatic left carotid stenosis (70% by North American Symptomatic Carotid Endarterectomy Trial criteria) who presented with right homonymous hemianopia compatible with a left posterior cerebral artery stroke. Arterial spin labeling demonstrates hypoperfusion throughout the left cerebral hemisphere (**a**) and CTA demonstrates a fetal left posterior cerebral artery supplied by the left internal carotid artery (**b**). Florbetaben PET/CT demonstrates no to sparse amyloid plaque (**c**). Flortaucipir PET/CT prior to the event demonstrates no areas of cortical tau retention (**d**). Flortaucipir PET/CT following the event (**e**) demonstrates focal tau retention in the left posterior cerebral artery territory (arrows), likely as a response to cerebral ischemia

Molecular imaging has a developing role in the evaluation of VaD. Hypometabolism in VaD is pronounced in structures spared in early AD including the anterior cingulate cortex, deep gray nuclei, primary cortices, and middle temporal gyrus, with Kerrouche et al. reporting 100% accuracy in separating VaD from AD [143, 144]. Amyloid imaging is less helpful in discriminating between VaD and AD, with at least a quarter of VaD patients demonstrating amyloid uptake and those that are PiB positive have similar distribution to AD [145–147]. Positive amyloid imaging, however, may predict worse cognitive function in VaD [146, 148]. While it is known tau deposition occurs following cerebral ischemia (Fig. 10), reports of tau tracers in the evaluation of VaD are lacking [149].

## Frontotemporal dementia

Frontotemporal dementia (FTD) or frontotemporal lobar degeneration (FTLD) is the second most common NDD in patients under 65 years of age, only behind AD [150]. The estimated prevalence is approximately 1–5 in 100,000 people [151, 152]. There is an equal predilection of the disease between men and women, most commonly diagnosed between the ages of 45 and 65; however, patients can be diagnosed as early as the second to third decade [150]. The most common subtype is the behavioral variant (bvFTD), characterized by social disinhibition, obsessive behaviors, and hyperorality, as well as the aptly named semantic (svPPA) and nonfluent/agrammatic primary progressive aphasias (nfPPA). Three distinct pathologic subtypes are described; first Pick reported argyrophilic cytoplasmic inclusions (of predominantly 3R tau) within cortical neurons leading to ballooning of the cells and eventually gliosis, later TAR DNA-binding protein 43 (TDP-43) and fused-in-sarcoma (FUS) pathologic variants were described [153, 154]. Heritable forms make up at least 10% of cases, including those with microtubule-associated protein tau (MAPT), Chromosome 9 Open Reading Frame 72 (C9ORF72), and Progranulin mutations [150, 155].

**Fig. 11:**
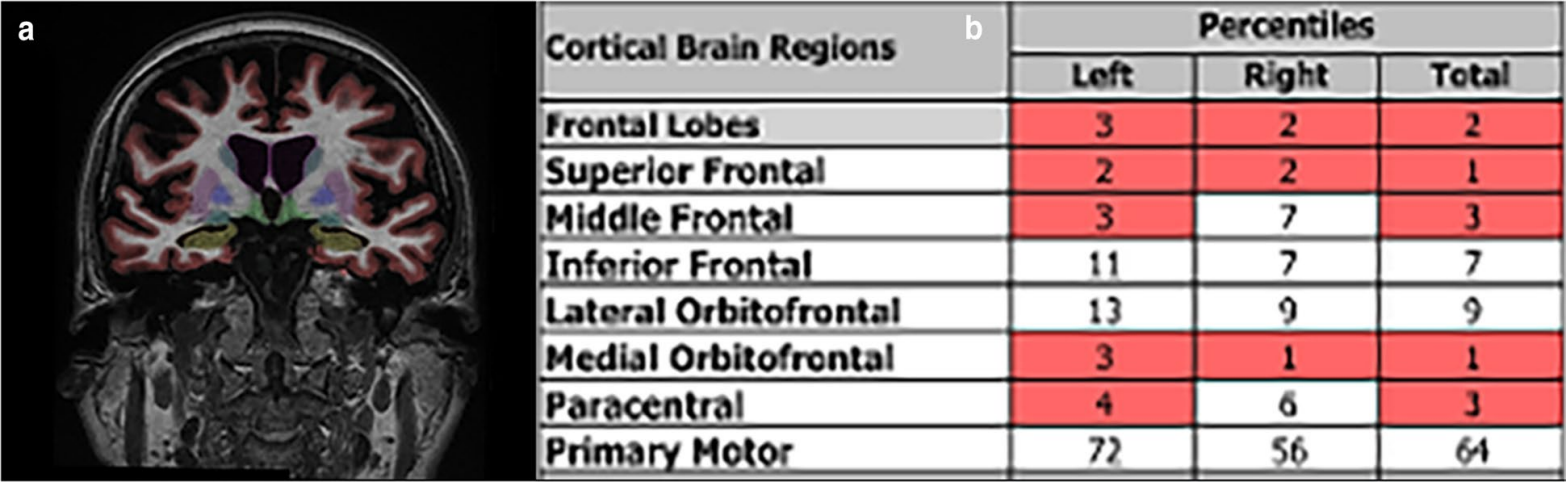
A 69-year-old female with odd behaviors including riding a children’s tricycle and impulsivity. Coronal volumetric T1-weighted images with proprietary automated segmentation software demonstrate volume loss in the frontal and temporal lobes (**a**). Volumetric quantitation identifies multiple regions in the frontal lobes in less than the fifth percentile of volume for age, including the medial orbital frontal lobes, typical of bvFTD (**b**)

As with the other neurodegenerative syndromes, volumetric T1-weighted imaging to assess for patterns of atrophy is commonly applied in FTD. Behavioral variant FTD tends to demonstrate marked atrophy involving the ventromedial prefrontal and insular cortices and anterior temporal lobes (Fig. 11), svPPA in the left inferior temporal lobe including the insular gyrus, and nfPPA in the posterior frontal, temporal, and parietal lobes [156]. Resting-state functional MRI has most consistently reported disruption of the salience network and frontoinsular and executive connections [157–159]. One study showed patients with FTD exhibit hypoperfusion on ASL in the frontal lobes and anterior cingulate cortex, with the hypoperfusion within the anterior cingulate cortex helping to differentiate FTD from AD [160]. Decreased stiffness by MRE in the temporal lobes has been reported [30].

**Fig. 12:**
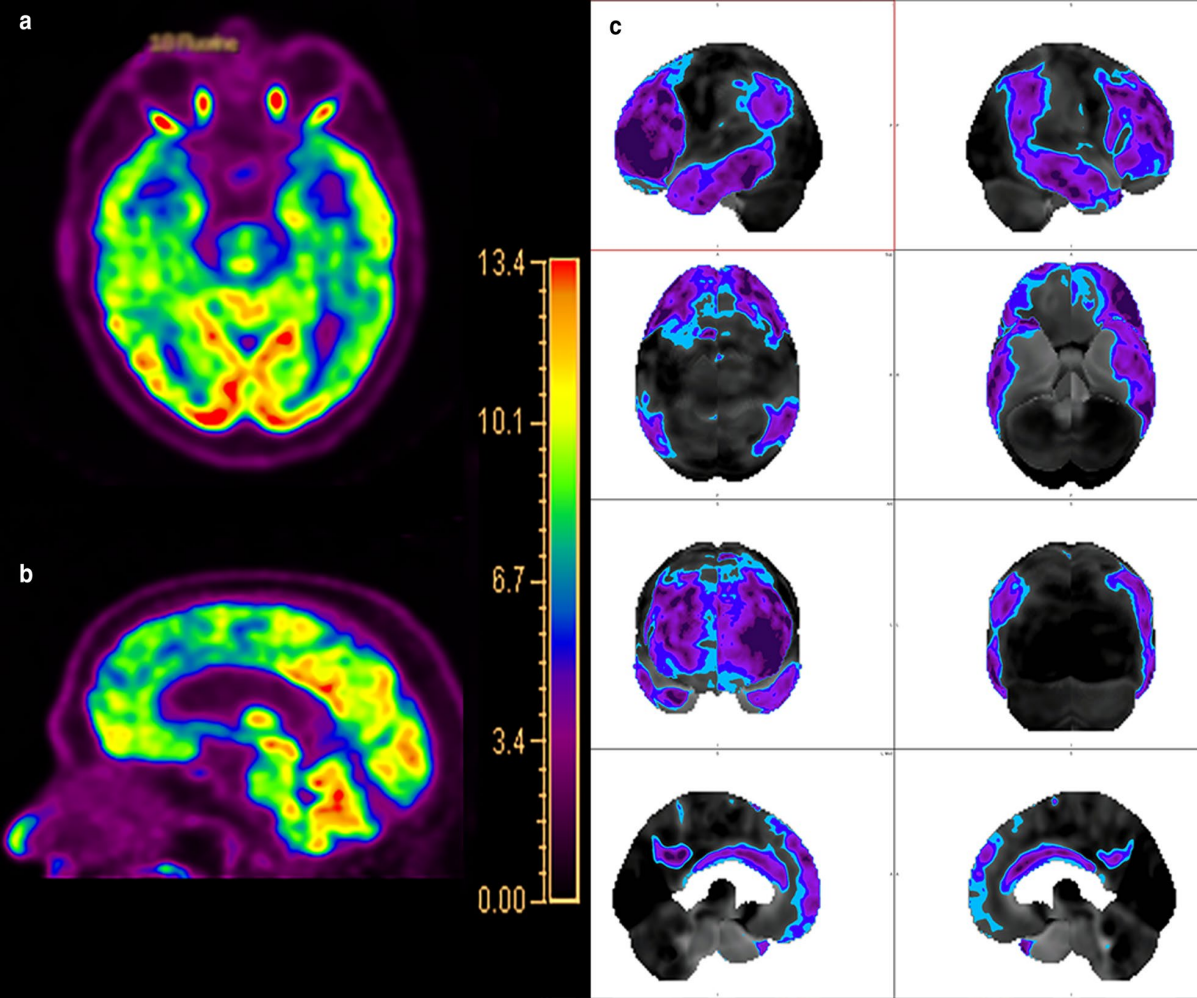
A 68-year-old man who presented to the memory care clinic with progressive personality changes and behavioral disturbances including violent outbursts and memory loss. Fluorodeoxyglucose PET/CT demonstrates marked frontal (**a**) and temporal (**b**) hypometabolism with relatively preserved parietal and occipital lobe activity including relative sparing of the PCC and precuneus. Single-subject statistical parametric mapping (**c**) from a similar patient demonstrates a similar pattern, with anterior greater than posterior cingulate hypometabolism, a hallmark of FTD, again with areas of blue progressing to purple representing further negative deviation from the mean uptake values of controls

**Fig. 13:**
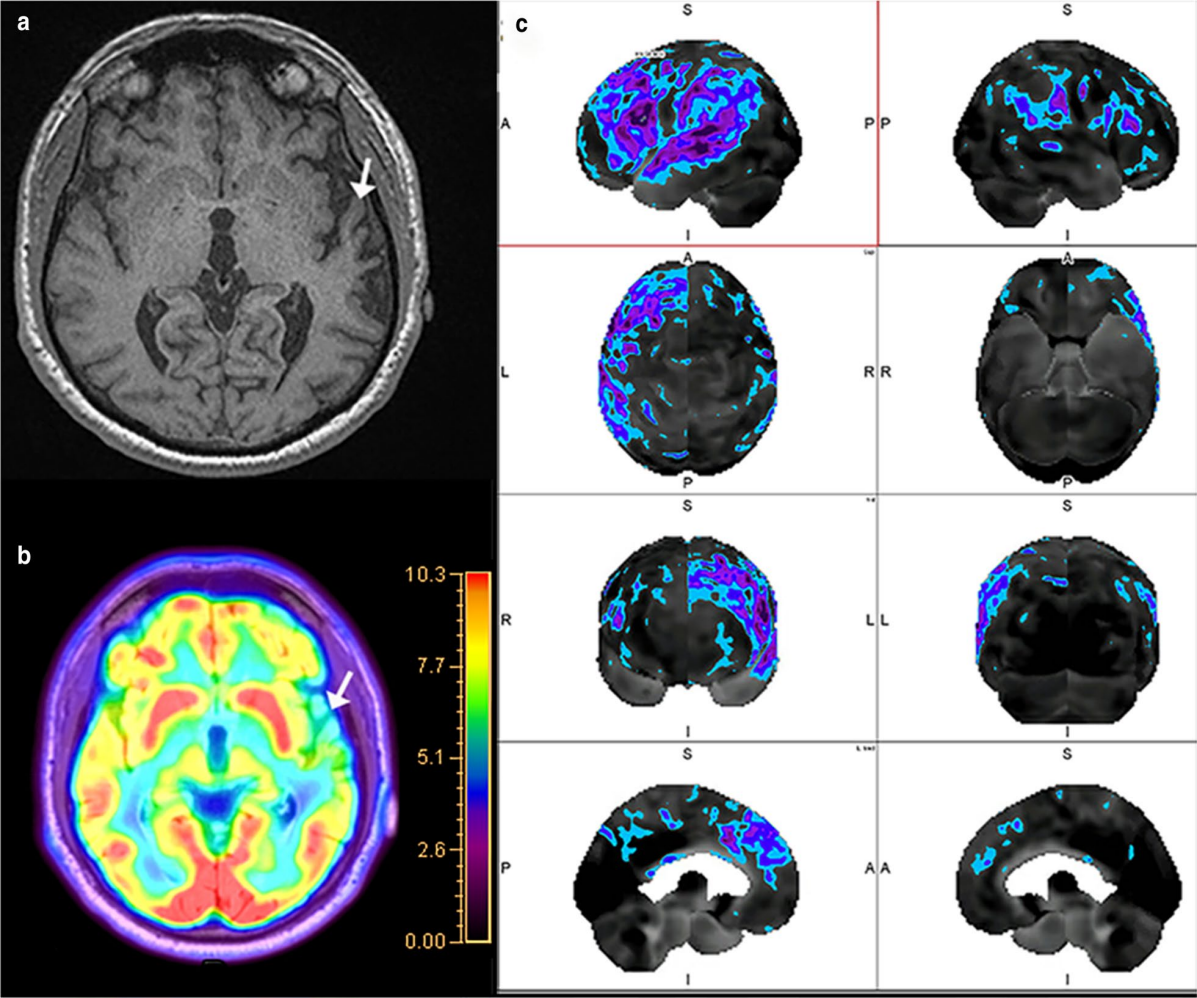
A 58-year-old man with notable agrammatism, impaired comprehension and repetition of syntactically complex sentences, and spared single word comprehension, Montreal Cognitive Assessment (MoCA) 16/30. T1-weighted volumetric image (**a**) demonstrates asymmetric atrophy of the left perisylvian and insular cortices (arrow). Fluorodeoxyglucose PET/MRI demonstrates hypometabolism in this region (arrow in **b**). Single-subject statistical parametric mapping of the FDG-PET/MRI (**c**) demonstrates the asymmetric left greater than right hypometabolism involving the frontal and temporal operculum and parietal lobes with sparing of the PCC and precuneus (as before, areas of blue progressing to purple represent further negative deviation from the mean uptake values of controls). Combining the clinical and imaging data, a diagnosis of nfPPA was rendered

Molecular imaging plays a crucial and ever-growing role in the evaluation of FTD. Unsurprisingly, hypometabolism corresponding to regions of atrophy in the frontal lobes, including the ventromedial region and anterior temporal lobes has been frequently reported in FTD (Fig. 12) [161, 162]. Fluorodeoxyglucose PET is also an important tool in distinguishing the PPA variants with ss-SPM demonstrating hypometabolism in the left or bilateral temporal poles, middle and inferior temporal gyri, and insula in svPPA and a more heterogeneous pattern of decreases in the inferior temporal gyrus, anterior cingulate cortex, and insula with sparing of the amygdala and hippocampi in nfPPA (Fig. 13) [40, 163]. Initial work on tau-labeled tracers in FTD has primarily been in patients harboring the MAPT mutation as they are known to have tau-related pathology; however, TDP-43-related syndromes have also demonstrated uptake with the basal and medial frontal lobes, inferior and lateral temporal lobes and temporal poles, and anterior cingulate cortex with the most involved regions varying by disease subtype [164, 165]. One study demonstrated co-localization between tau binding and microglial activation [166]. Lack of uptake of amyloid labeled radiotracers can differentiate FTD from AD [167]. Table 2 describes the common imaging features of Typical AD, DLB, VaD, and FTD.

**Table 2:** Synopsis of imaging features of common neurodegenerative disorders

	Typical AD	DLB	VaD	FTD
sMRI	Mesial temporal lobe atrophy	Frontotemporal atrophy	Two or more large territory, three or greater lacunar infarcts, or strategically placed infarcts. Involvement of cerebral hemisphere by greater than 25% WMHs	bv—Ventromedial prefrontal and insular cortical atrophy svPPA—left inferior temporal lobe including the insular gyrus nfPPA—posterior frontal, temporal, and parietal lobes
SW/T2*		Swallow tail sign	Microhemmorhages can be present	
MRS	Decreased NAA/myoinsitol in the PCC			
DTI	Decreased FA and increased MD in mesial temporal lobe, PCC, SLF	Decreased FA parieto-occipital lobes, PCC, striatum	Reduced FA and increased MD in the centrum semiovale and anterior periventricular white matter	Reduced FA in the UF, genu of the CC, and left ACC
rsfMRI	Hypoconnectivity of DMN	Hypoconnectivity of DMN and visual networks	Hypoconnectivity of DMN	Hypoconnectivity of salience network and disruption of frontoinsular and executive connections
ASL	Hypoperfusion involving the temporopartietal lobes			Hypoperfusion of the frontal lobes and anterior cingulate cortex
FDG-PET	Temporopartietal hypometabolism	Temporopartietal and occipital hypometabolism Cingulate island sign Occipital tunnel sign	Hypometabolism of the anterior cingulate cortex, deep gray nuclei, primary cortices, and middle temporal gyrus	bv—hypometabolism of ventromedial frontal lobe and anterior temporal lobes svPPA—hypometabolism of left or bilateral temporal poles, middle and inferior temporal gyri, and insula nfPPA—hypometabolism of the inferior temporal gyrus, anterior cingulate cortex, and insula with sparing of the amygdala and hippocampi
Amyloid PET	Often diffuse cortical uptake	Majority demonstrate uptake, primarily temporoparietal with more involvement of the occipital lobes compared to AD	At least 25% demonstrate uptake	Generally lack of uptake
Tau PET	Uptake within the temporopartietal neocortical areas—in vivo Braak staging	Inferolateral temporal and parietal lobes with less mesial temporal lobe activity compared to AD		Uptake in the inferior and lateral temporal lobes and temporal poles, and anterior cingulate cortex
DAT/Dopamine imaging		Decreased striatal uptake	Striatal infarcts can lead to decreased uptake	

*sMRI* structural magnetic resonance imaging, *MRS* Magnetic resonance spectroscopy, *DTI* Diffusion tensor imaging, *rsfMRI* resting-state functional magnetic resonance imaging, *ASL* Arterial spin labeling, *FDG* Fluorodeoxyglucose, *PET* Positron emission tomography, *DAT* Dopamine transporter, *AD* Alzheimer’s disease, *NAA* N-acetylaspartate, *FA* Fractional anisotropy, *MD* Mean diffusivity, *DMN* Default mode network, *PCC* Posterior cingulate cortex, *SLF* Superior longitudinal fasciculus, *DLB* Dementia with Lewy bodies, *VaD* Vascular dementia, *WMH* White matter hyperintensity, *FTD* Frontotemporal dementia, *bv* behavioral variant, *PPA* Primary progressive aphasia, *sv* semantic variant, *nf* nonfluent, *UF* Uncinate fasciculus

## Parkinson’s disease and parkinsonian syndromes

Parkinsonian disorders are a heterogeneous group of syndromes sharing the clinical features of extrapyramidal symptoms and bradykinesia and common pathology related to alpha-synuclein and tau with degeneration of the nigrostriatal pathway. These conditions afflict at least 1% of the worldwide population greater than 70 years of age [168]. The differences between Parkinson’s disease and DLB have been previously discussed in the DLB section. Three of the most relevant parkinsonian syndromes will be reviewed; multisystem atrophy (MSA), progressive supranuclear palsy (PSP), and corticobasal degeneration (CBD).

**Fig. 14:**
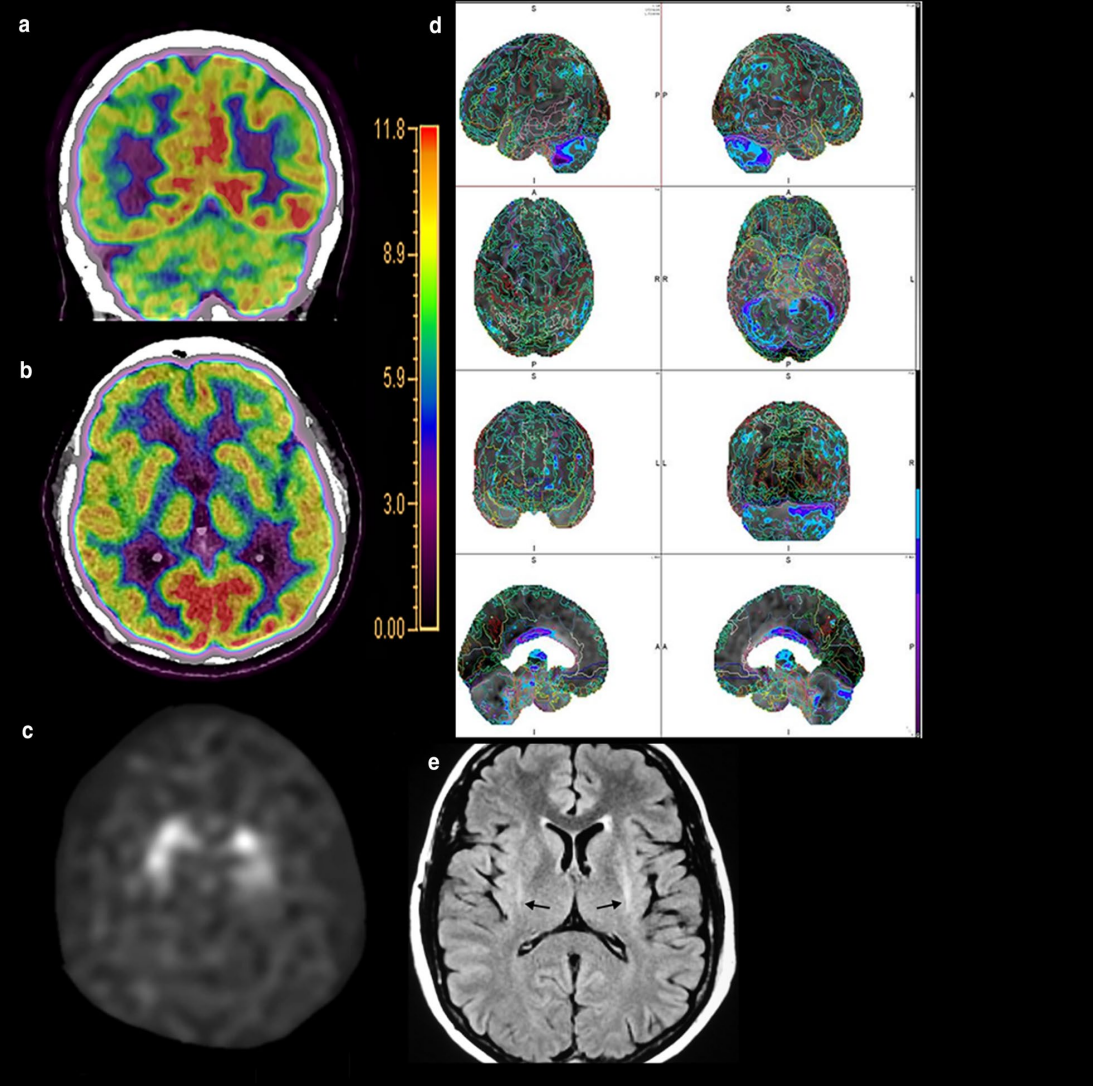
A 76-year-old man with a history of memory impairment and parkinsonism including gait and proprioceptive dysfunction. Fluorodeoxyglucose PET/CT demonstrates hypometabolism in the right greater than left cerebellar hemispheres (**a**) and bilateral striatum (**b**), best appreciated on ss-SPM (areas of blue and purple representing negative deviation in uptake from controls, **d**). Iodine-123-FP-CIT scan shows left greater than right primarily putaminal presynaptic neuronal degeneration (**c**). Findings are compatible with MSA-P. Axial T2 FLAIR image of a separate patient demonstrates the lateral putaminal rim sign of hyperintensity (arrows in **e**)

**Fig. 15:**
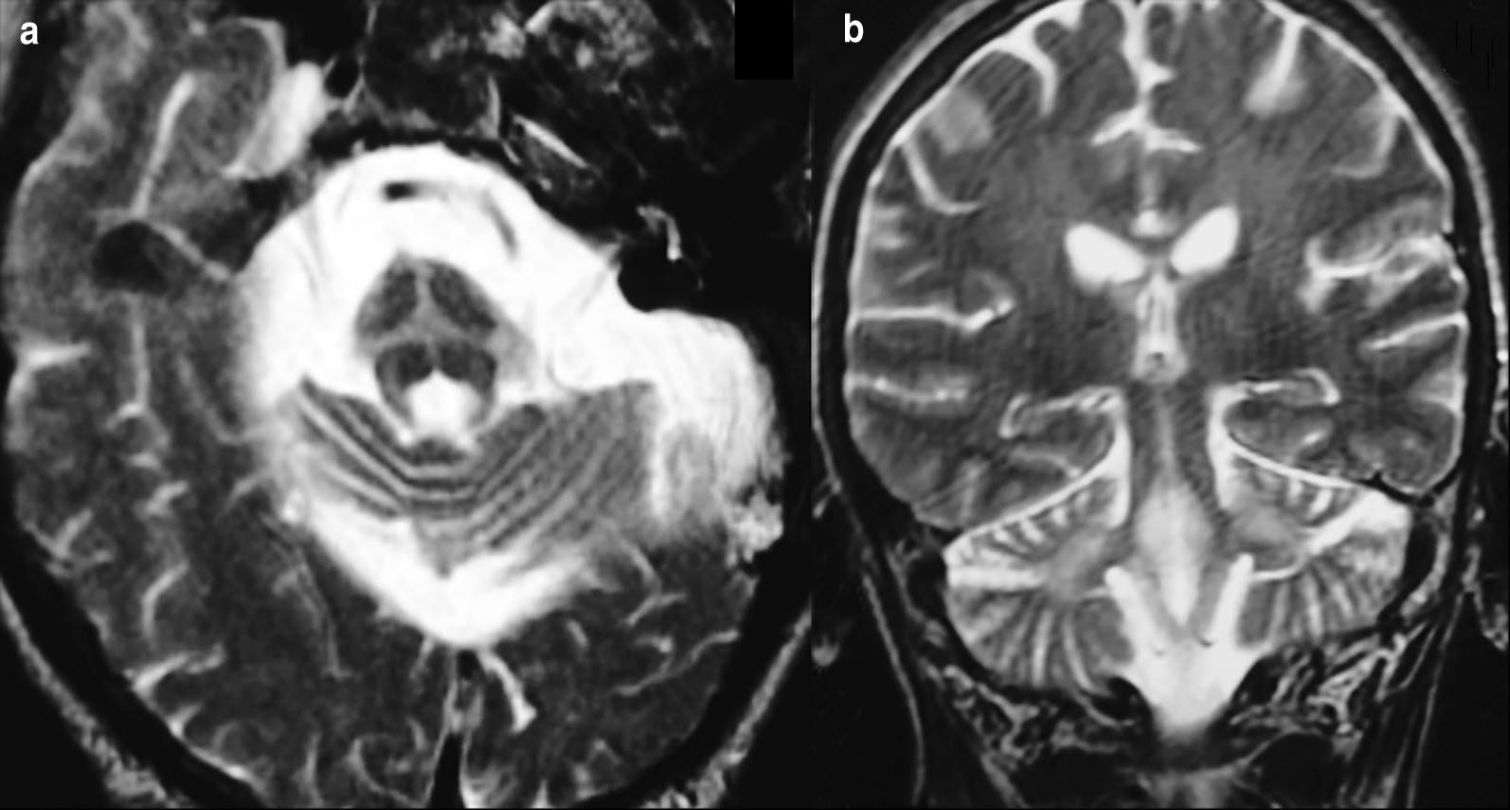
Axial T2-weighted image demonstrates cruciform hyperintense signal in the pons, the so-called hot cross bun sign of MSA-C (**a**). Coronal T2-weighted image at the level of the brainstem demonstrates hyperintense signal in the bilateral brachis pontis, another common finding in the disease (**b**)

*Multisystem atrophy* is a neurodegenerative disorder with multiple subtypes characterized clinically by parkinsonism with varying cerebellar, autonomic, and pyramidal dysfunction sharing common alpha-synucleinopathy pathology [169]. Two main subtypes exist, one with dominant parkinsonian features (MSA-P), and one with dominant cerebellar dysfunction (MSA-C) [170]. On structural 1.5 T MRI, MSA-P has been reported to have a characteristic appearance of a lateral rim of T2/proton density-weighted hyperintensity adjacent to the putamen and T2 hypointense signal involving the dorsolateral putamen, although the value of this finding has been questioned at higher field strengths (Fig. 14) [171, 172]. The hallmark imaging feature seen in up to 80% of patients with MSA-C is cruciform T2 hyperintense signal within the basis pontis, the so-called hot cross bun sign, although this imaging finding can also be present in spinocerebellar ataxia syndromes (Fig. 15) [173]. Additional structural imaging features of MSA-C include atrophy and T2 hyperintense signal within the pons, medulla, middle cerebellar peduncles, and cerebellar hemispheres [174, 175]. Diffusion tensor imaging exhibits widespread microstructural alterations when compared to controls including increased MD and reduced FA in the superior, middle, and inferior cerebellar peduncles and in the corona radiata and commissural fibers [176]. Patterns of reduced MTR have been shown to help differentiate MSA from PD and PSP [177]. Hypoconnectivity in the DMN, sensorimotor network, visual association cortices, and cerebellum has been reported in MSA-C [178]. Within the same study hypoperfusion of the cerebellum was exhibited on ASL imaging [178]. On FDG-PET MSA is distinguished by hypometabolism involving the bilateral putamina and cerebellar hemispheres, with the cerebellar hypometabolism reported in both MSA-C and MSA-P, with a sensitivity of 76% and specificity of 98% for the diagnosis by visual interpretation (Fig. 14) [179]. While MSA shows reduced uptake within the putamina on I-123-FP-CIT SPECT scans, this was not shown to correlate with disease severity [180].

**Fig. 16:**
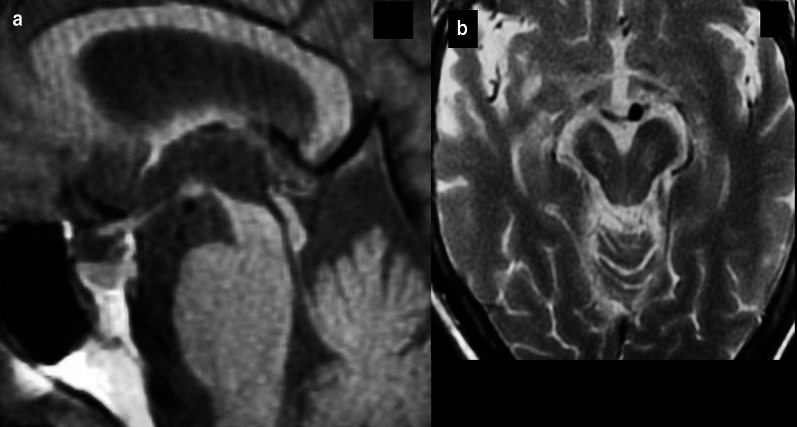
Sagittal T1-weighted image in the midline coned to the brainstem demonstrates midbrain atrophy creating the so-called hummingbird sign of PSP (**a**). Axial T1-weighted image at the level of the midbrain demonstrates decrease in the AP dimension of the midbrain and widening of the interpeduncular cistern; the so-called morning glory sign (**b**)

*Progressive supranuclear palsy* is clinically defined by parkinsonism with vertical supranuclear gaze palsy and prominent postural instability with falls within the first year of onset and pathologically by 4R tau isoform NFTs in the basal ganglia, diencephalon, and brainstem [181, 182]. As with MSA, characteristic patterns of brainstem atrophy are present including reduced anterior–posterior dimension of the midbrain and widening of the interpeduncular cistern leading to the “mickey mouse sign” on axial and the “hummingbird sign” on sagittal images (Fig. 16) [183, 184]. Reduced FA in the posterior frontal lobes and cerebellar peduncles was reported to have a specificity of 91–96% and sensitivity of 85–95% in differentiating PSP from DLB [185]. Hypoconnectivity on rsfMRI has been documented of the lateral visual, auditory, cerebellar, and insular networks [186]. Typical findings of FDG-PET include hypometabolism of the brainstem and midline frontal cortex with a reported sensitivity of 60% and specificity of 96% by visual interpretation with significant augmentation of sensitivity when ss-SPM is added [179]. 18-Fluorine-PI-2620, which selectively binds to the 4R tau isoform, has shown promise as a biomarker in PSP, though further evaluation is warranted [187]. Single-photon emission computed tomography imaging of pre- and postsynaptic striatal neuronal degeneration shows similar patterns to and is not able to reliably differentiate PD from PSP [188].

**Fig. 17:**
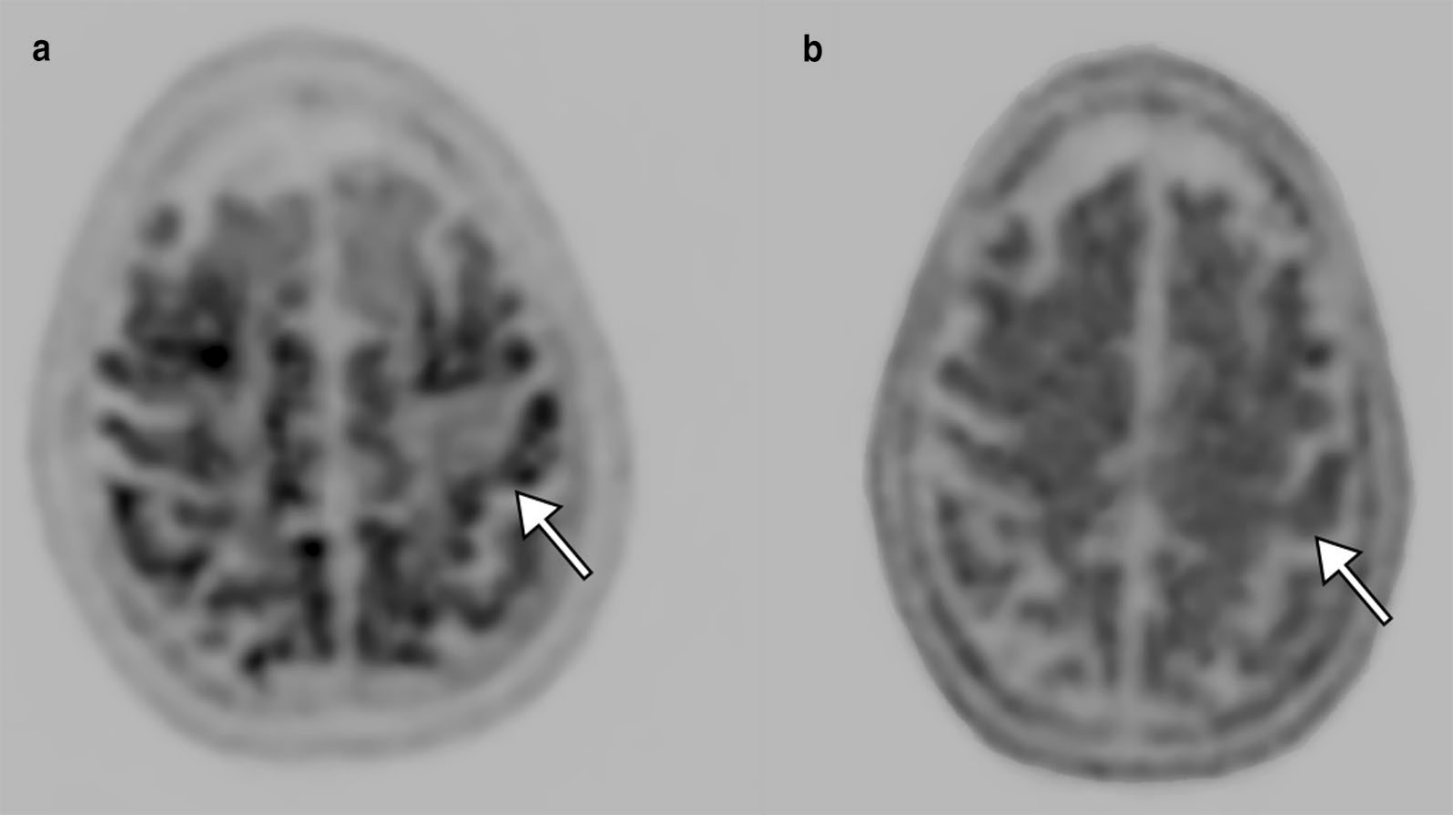
A 60-year-old female with cognitive decline (MoCA 19/30) and limb apraxia. Flortaucipir PET/CT (**a**) demonstrates tau retention in the bilateral perirolandic regions (arrow indicating left precentral gyrus). Florbetapir PET/CT (**b**) demonstrates radiotracer activity in the bilateral perirolandic regions (arrow indicating left precentral gyrus). Final diagnosis was CBS secondary to underlying AD, given the abnormal amyloid binding

*Corticobasal degeneration* is a specific form of the corticobasal syndrome (CBS) defined by extrapyramidal symptoms which are often asymmetric including an “alien limb” phenomenon with later onset loss of multidomain cognitive functioning with preserved episodic memory and poor response to levodopa therapy [189, 190]. Pathologically CBD is primarily a 4R tauopathy which demonstrates ballooned neurons in many areas of brain including the primary cortices as well as the cingulate gyrus, amygdala, insular cortex, and claustrum [191]. Typical structural MRI findings include asymmetric atrophy of the posterior frontal and parietal lobes contralateral to the patient’s symptoms with associated atrophy of the contralateral cerebral peduncle and subcortical T2 FLAIR hyperintensity with relative preservation of basal ganglia volume and signal [174, 175, 192]. Reduced FA has been reported in the pre- and post-central gyri, cingulum, and supplementary motor area [193]. Resting-state functional MRI has documented hypoconnectivity of the lateral visual and auditory networks and hyperconnectivity of the salience and executive control networks [186]. Fluorodeoxyglucose PET shows similar findings with hypometabolism involving the primary cortices and additionally basal ganglia contralateral to the affected side [179, 194, 195]. Early research on Tau tracers for CBD has shown mixed results with 18-F-AV-1451, 18-F-PI-2620 may be a more appropriate tracer given its affinity for the 4R tau isoform [196–198]. The role of amyloid labeled tracers is uncertain given the overlap of CBS secondary to other dementias and imperfect tau isoform selectivity of current radiotracers (Fig. 17) [198]. Iodine-123-FP-CIT SPECT demonstrates asymmetric decreased striatal uptake with less disproportionate involvement of the putamen when compared to other Parkinsonian syndromes [199, 200]. Table 3 describes the common imaging features of parkinsonian syndromes.

**Table 3:** Synopsis of imaging features of parkinsonian syndromes

	CBD	PSP	MSA
sMRI	Asymmetric atrophy of the posterior frontal and parietal lobes and cerebral peduncle contralateral to symptoms	“Mickey mouse” and “hummingbird” signs secondary to brainstem atrophy	MSA-p—lateral rim of T2/PD hyperintensity adjacent to putamen (1.5 T) MSA-c—Hot cross bun sign, brainstem and cerebellar atrophy and T2 hyperintensity
SW/T2*			
MRS			
DTI	Reduced FA in the pre- and post-central gyri, cingulum, and SMA	Reduced FA in the posterior frontal lobes and cerebellar peduncles	Reduced FA in the superior, middle, and inferior cerebellar peduncles and in the corona radiata and commissural fibers
rsfMRI	Hypoconnectivity of the lateral visual and auditory networks and hyperconnectivity of the salience and executive control networks	Hypoconnectivity of the lateral visual, auditory, cerebellar, and insular networks	Hypoconnectivity of the DMN, sensorimotor network, visual associated cortices, and cerebellum
ASL			Hypoperfusion of the cerebellum
FDG-PET	Hypometabolism involving the primary cortices and additionally basal ganglia contralateral to the affected side	Hypometabolism involving the brainstem and midline frontal cortex	Hypometabolism involving the bilateral putamina and cerebellar hemispheres
Amyloid PET	May show uptake in CBS (not pure CBD) secondary to underlying amyloid-related NDD		
Tau PET	Uptake of 18-F-PI-2620 in frontoparietal cortices and basal ganglia structures contralateral to affected side	Uptake of 18-F-PI-2620 in the basal ganglia and brainstem structures	
DAT/Dopamine imaging	Asymmetric decreased striatal uptake caudate relatively more impacted than putamen	Decreased striatal uptake	Reduced uptake within the putamina

*sMRI* structural magnetic resonance imaging, *MRS* Magnetic resonance spectroscopy, *DTI* Diffusion tensor imaging, *rsfMRI* resting-state functional magnetic resonance imaging, *ASL* Arterial spin labeling, *FDG* Fluorodeoxyglucose, *PET* Positron emission tomography, *DAT* Dopamine transporter, *MSA-p* Multisystem atrophy, Parkinsonian type, *PD* Proton density, *MSA-c* Multisystem atrophy cerebellar type, *DMN* Default mode network, *FA* Fractional anisotropy, *PSP* Progressive supranuclear palsy, *CBD* Corticobasal degeneration, *SMA* Supplementary motor area, *CBS* Corticobasal syndrome, *NDD* Neurodegenerative disease

## Miscellaneous syndromes

### Cerebral amyloid angiopathy

**Fig. 18:**
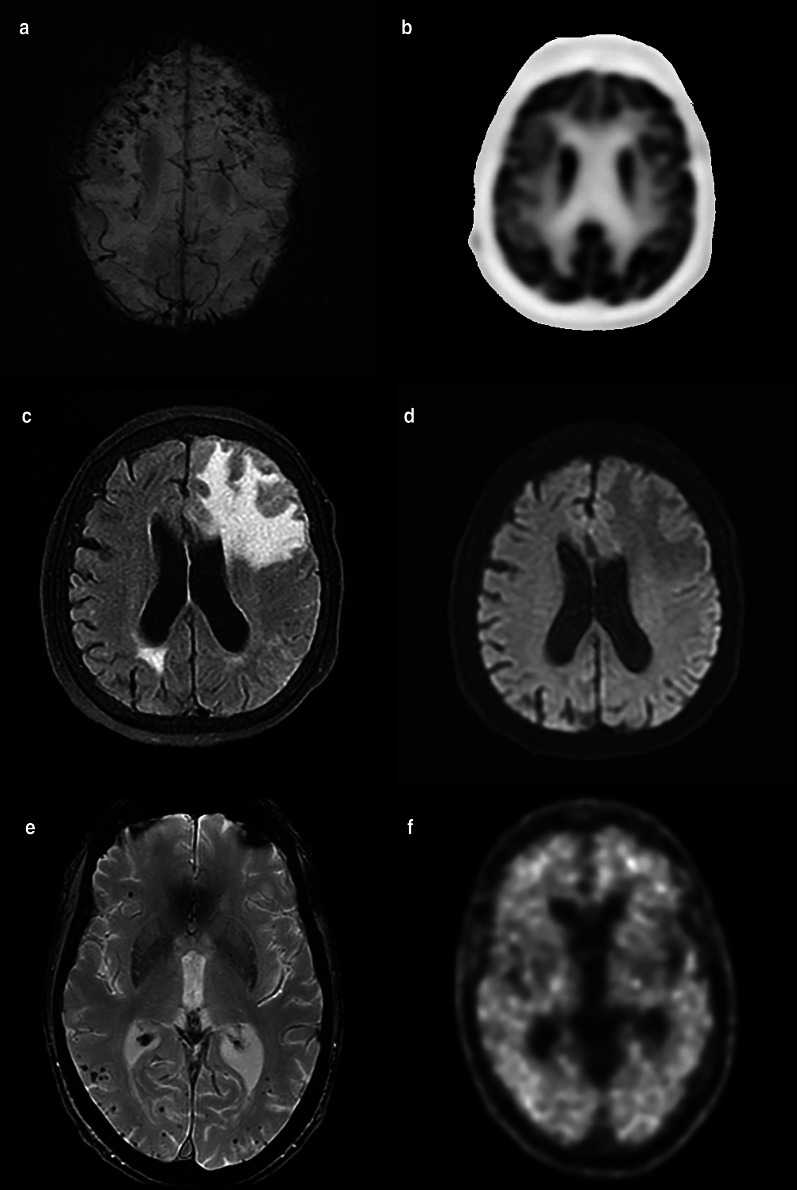
A 81-year-old female with a gradual decline in short term memory and depression. Susceptibility-weighted image demonstrates numerous subcortical microhemorrhages (**a**). Fluorodeoxyglucose PET/MRI (**b**) demonstrates relatively normal cerebral metabolism. Later she presented with an acute worsening of symptoms including new right-side weakness and dysphasia. Magnetic resonance imaging demonstrated T2 FLAIR hyperintensity within the left frontal lobe (**c**) without restricted diffusion (**d**). Final diagnosis was amyloid beta-related angiitis. Cognitive function improved following administration of steroids. Fluorodeoxyglucose PET/MRI suggested against concurrent AD. Images from a separate patient demonstrate occipito-temporal subcortical microhemorrhages (**e**) with diffuse amyloid uptake on florbetapir PET/CT, a pattern suggesting concurrent CAA with AD (**f**)

Cerebral amyloid angiopathy (CAA) is a disease of Aβ deposition in the walls of primarily the small cortical and leptomeningeal arteries and arterioles favoring the posterior regions [201, 202]. Approximately 10–20% of intracerebral hemorrhage at autopsy may be attributed to CAA with up to 80% of patients with CAA demonstrating concurrent AD pathology [203]. The updated Boston criteria 2.0, which includes findings on structural MRI of at least two either lobar or subcortical hemorrhagic lesions or scattered superficial siderosis or one of the aforementioned hemorrhagic lesions with greater than 20 perivascular spaces or white matter hyperintensities in one hemisphere without evidence of deep hemorrhagic lesions or evidence of other cause, was reported to have 64.5% sensitivity and 95% specificity for the diagnosis of typical CAA [204]. There are two rarer subtypes of CAA, inflammatory CAA, characterized by lobar edema with overlying leptomeningeal enhancement and subcortical microhemorrhages without diffusion restriction and amyloidoma, characterized by a solitary enhancing mass with surrounding edema (Fig. 18) [205]. One study demonstrated amyloid PET has good sensitivity to discriminate CAA from controls, however, with modest specificity (sensitivity of 91% and specificity of 55%) [206]. The poor specificity of amyloid PET for the diagnosis of CAA is attributed to the overlap with AD pathology; the posterior predominance of CAA has led to reports of the occipital-to-posterior cingulate or global ratio as a possible means of discrimination from AD [207, 208]. Fluorodeoxyglucose PET has demonstrated similar findings with decrease in SUVr ratio of the occipital lobe relative to the PCC in patients with CAA compared to AD [209]. As with AD, early reports of tau agents in CAA have demonstrated increased binding correlated with worsening cognition, which was not demonstrated with amyloid agents [210].

### Chronic traumatic encephalopathy

**Fig. 19:**
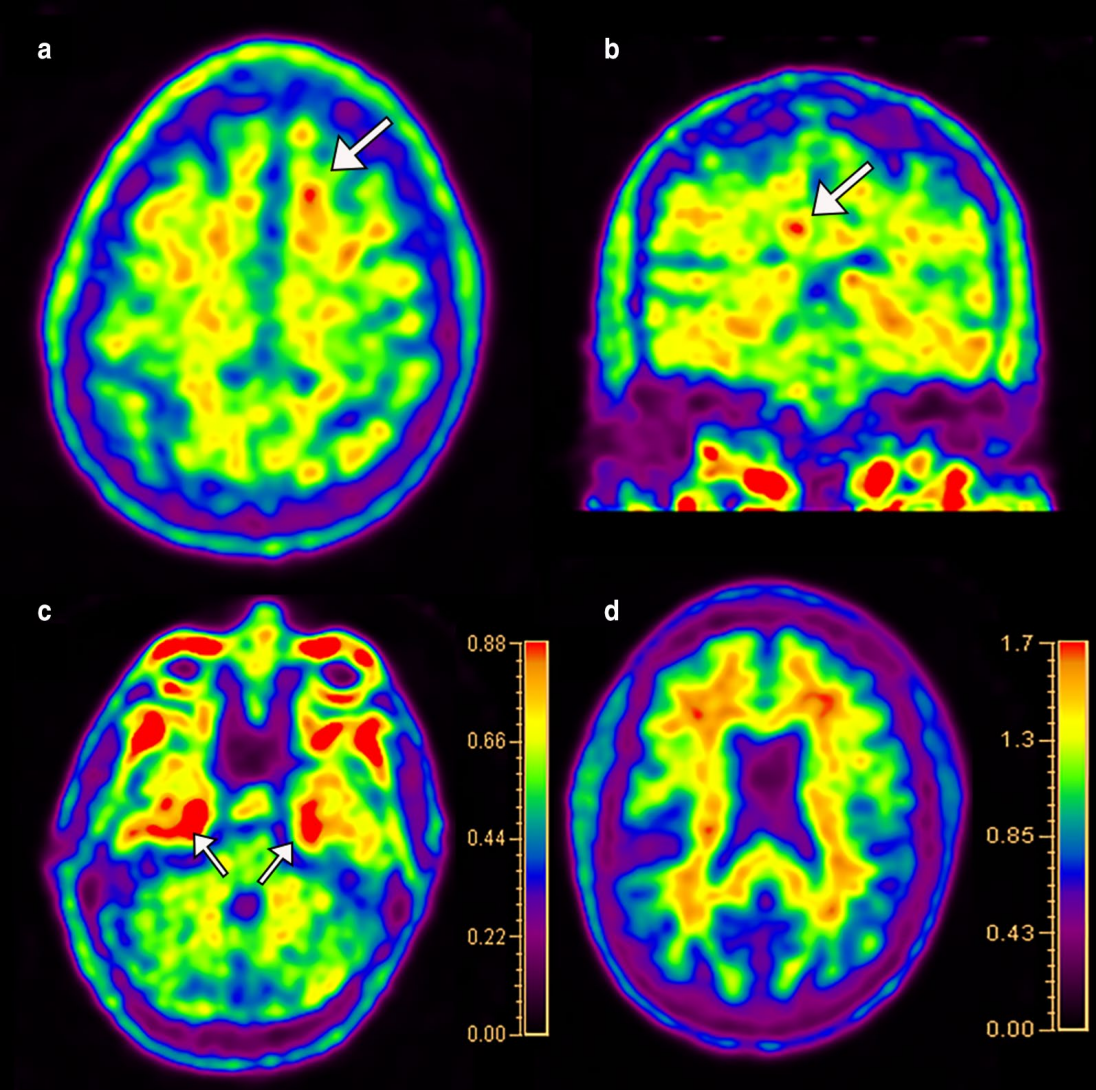
A 70-year-old man with history of military combat and traumatic brain injury with MCI, MoCA 26/30. Flortaucipir PET/CT demonstrates regions of tau retention in the sulcal depths of the parasagittal frontal cortices (arrows in **a** and **b**). Tau retention is also noted in the bilateral mesial temporal lobes (arrows in **c**). Florbetapir PET/CT (**d**) demonstrates no significant cortical amyloid uptake involving the parietal lobes. Given the findings on PET/CT memory impairment was diagnosed as related to CTE

Chronic traumatic encephalopathy (CTE) is a tauopathy secondary to repetitive mild traumatic brain injury more recently described in the literature with clinical features of decreased attention and memory, affective disturbances, psychosis, and gait and speech difficulties [211, 212]. Four progressive pathologic stages of the disease are defined with perivascular hyperphosphorylated tau NFTs beginning in the dorsolateral frontal cortices and spreading to the temporal and parietal lobes and deep nuclei with sparing of the calcarine cortex except in severe cases [213]. Amyloid plaques are not considered a feature of the disease [211]. Structural imaging shows an exceedingly higher than expected number of patients with a cavum septum pellucidum, felt to be related to shear forces due to a CSF fluid wave from trauma [213, 214]. Additionally, generalized cerebral white and gray matter volume loss has been reported [214, 215]. Reduced FA in white matter tracts including the superior and inferior longitudinal fasciculus, corona radiata, cerebral peduncle, uncinate fasciculus, and anterior thalamic radiations as well as the ventral striatum has been described [216–218]. Tau radiotracers are reported to have increased uptake in the frontotemporal lobes including the medial temporal lobes with frontal-lobe predominant involvement of the sulcal depths (Fig. 19) [219, 220]. Areas of hypometabolism on FDG-PET generally mirror the regions of tau retention [215, 220]. Variable positivity has been seen with amyloid tracers [219].

### Amyotrophic lateral sclerosis

Amyotrophic lateral sclerosis (ALS) is a progressive neurodegenerative disorder characterized primarily by upper and lower motor neuron degeneration leading to clinical signs of progressive weakness, muscle atrophy, and fasciculations starting in the limbs [221]. The pathologic hallmark of the disease is ubiquitinated TDP-43 inclusions [222]. T2 hyperintensity involving the corticospinal tracts (CSTs) was one of the earliest reported imaging features of ALS; however, studies have demonstrated inconsistency in this finding [223–225]. Reduced FA on DTI and NAA on MR spectroscopy in the CSTs has been consistently reported [226, 227]. Increased iron deposition quantified by T2* imaging has been shown in the motor cortex of patients with ALS [228]. Resting-state functional MRI shows decreased connectivity in the motor network [229]. Fluorodeoxyglucose PET imaging has demonstrated hypometabolism in the motor/perirolandic and frontal cortices as well as the occipital lobes with a sensitivity of 94.8–95.4% and specificity of 80–82.5% for the diagnosis [230, 231]. Binding in the CSTs of 2-([1E,3E]-4-[6-([11C]methylamino)pyridinyl]buta-1,3-dienyl)benzo[d]thiazol-6-ol ([11C]PBB3), a tau radiotracer, was shown to correlate with upper motor neuron signs in a patient with ALS/parkinsonian dementia complex overlap; however, extensive reports of tau agents are lacking at the current time, although an attractive target given the pathologic findings of TDP-43 in ALS [222, 232].

### Huntington’s disease

**Fig. 20:**
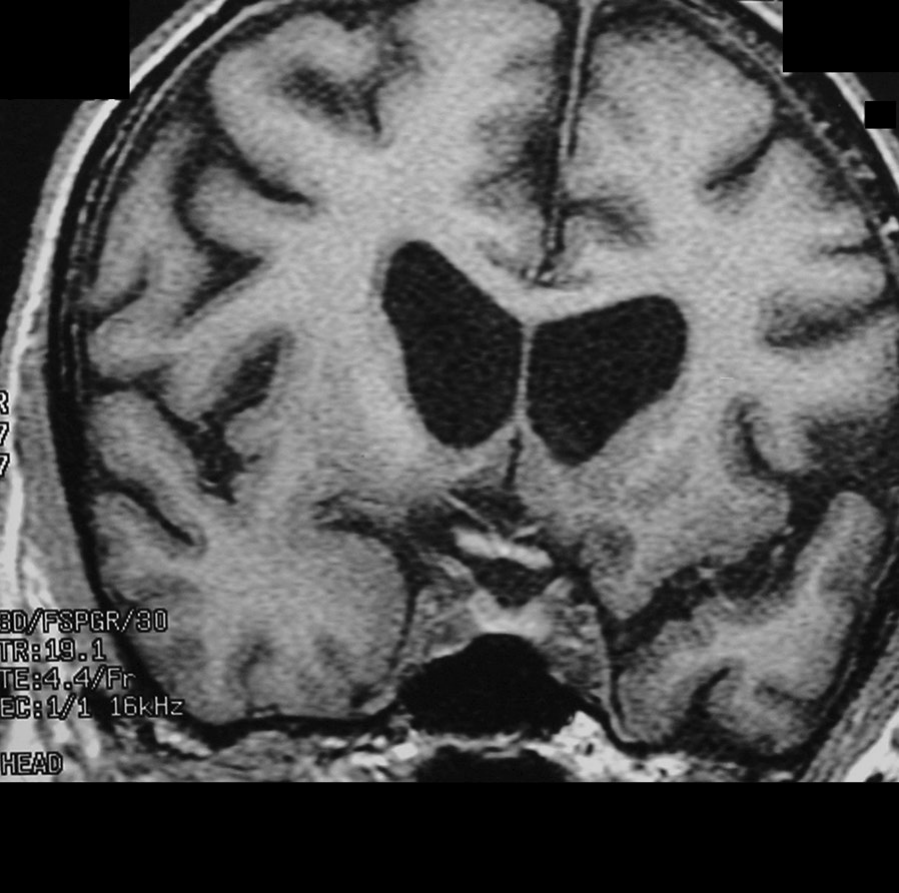
Coronal T1-weighted image demonstrates atrophy of the bilateral caudate heads with “box-shaped” ventricles

Huntington’s disease is an autosomal dominant neurodegenerative disorder characterized clinically by chorea, dementia, and psychosis, genetically by CAG trinucleotide repeat expansion on chromosome 4, and pathologically by atrophy in the striatum with involvement of other subcortical and cortical regions at higher stages [233]. Structural imaging shows striking atrophy of the striatum with ex vacuo dilation of the frontal horns of the lateral ventricles (so-called box-shaped ventricles), which is predictive of symptom onset (Fig. 20) [234]. Studies have overall demonstrated increased FA, felt to be related to selective neuronal degeneration (i.e. as selected tracts are damaged the remaining fibers demonstrate more organization), and increased MD in the basal ganglia [235]. Additional regions with reduced FA and increased MD include the CSTs and corpus callosum [235]. Hypometabolism in the striatum as well as the frontal and temporal lobes has been reported, with striatal hypometabolism a potential marker of time to symptom onset [236]. Decreased striatal dopamine receptor binding, most commonly utilizing [11C]raclopride-PET, has been exhibited in HD patients [237]. Other research targets have included adenosine, cannabinoid, and gamma-aminobutyric acid (GABA) receptors (diminished in HD), microglial activation (increased), phosphodiesterase 10A enzymatic activity (decreased), and synaptic vesicle protein 2A expression (decreased), all of which are not yet utilized clinically [238]. To date, a radiotracer targeting the Huntington protein is not available; however, this would obviously be an attractive target.

### Creutzfeldt–Jakob disease

**Fig. 21:**
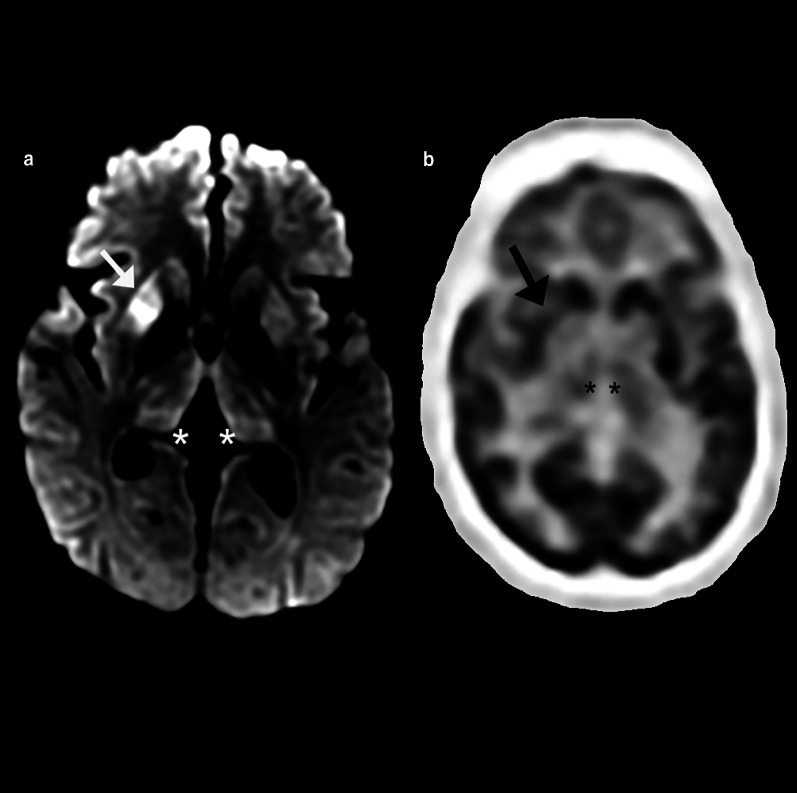
A 64-year-old female with three months of progressive functional decline, failure to thrive, and weakness. Diffusion-weighted imaging (**a**) demonstrates hyperintensity in the right caudate head and lentiform nucleus (arrow) and the bilateral thalami with an L-shaped or “hockey stick” configuration (asterisks). Fluorodeoxyglucose PET/MRI (**b**) demonstrates corresponding asymmetric hypometabolism in the right lentiform nucleus (arrow) and bilateral thalami (asterisks). Cerebrospinal fluid 14-3-3 protein was positive and pathological examination of the brain was consistent with CJD

Creutzfeldt–Jakob disease (CJD) is a rapidly progressive uniformly fatal prion-driven NDD afflicting approximately 1–2 persons per million worldwide, with three subtypes, the sporadic subtype representing the majority of cases [239]. Characteristic MRI features include hyperintensity on T2 or diffusion-weighted imaging (DWI) involving the cortex (cortical ribboning) and basal ganglia with involvement of the thalamus (“hockey stick sign”) less commonly reported in the sporadic subtype and more common in variant CJD (Fig. 21) [240]. T1 hyperintensity in the globus pallidus, thought to be related to accumulation of misfolded proteins, may also be observed, even without DWI changes [241]. Decreased NAA on MR spectroscopy has been reported and is likely related to neuronal death [242]. Asymmetric hypometabolism in the frontal and parietal cortices with or without decreased activity in the basal ganglia has been demonstrated with FDG-PET (Fig. 21) [243, 244]. Sporadic case reports have shown no significant amyloid and F-18 flortaucipir retention with one case demonstrating uptake of 18F-THK5351, felt to be related to off-target binding of MAO-B activation in astrocytosis and correlating to the areas of signal abnormality [245–248].

### Pseudodementia

**Fig. 22:**
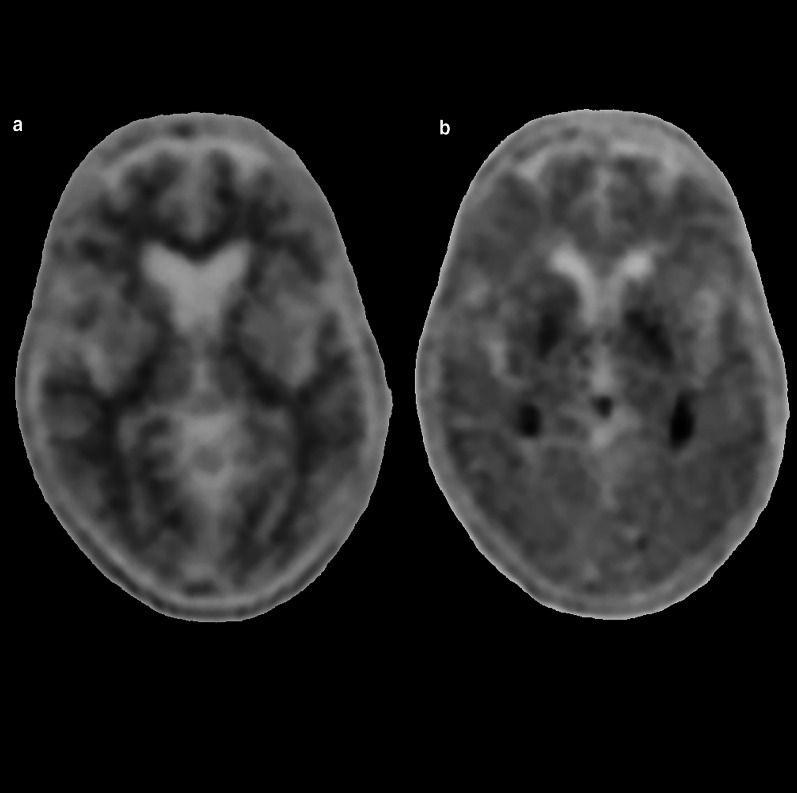
A 65-year-old female with reported gradual decline in short term memory and depressive symptoms, initial MoCA 25/30. Florbetapir PET/CT demonstrates normal white matter distribution with no regions of abnormal cortical uptake, compatible with no to sparse amyloid plaque (**a**). Flortaucipir PET/CT demonstrates no cortical tau retention with off-target binding in the bilateral choroid plexuses and basal ganglia (**b**). Score on MoCA improved to 29/30 on antidepressant therapy. Final diagnosis was pseudodementia related to major depressive disorder

Technically a non-NDD, pseudodementia was first described in 1961 by Kiloh as a condition of apparent dementia most commonly to a secondary mental illness such as depression leading to memory loss of recent and remote events and inattention [249, 250]. Often a geriatric depression scale is performed with initial memory care clinic consultation to exclude concomitant mental illness driving dementia symptoms [251]. Molecular imaging provides an excellent method to discriminate non-NDDs/pseudodementia from NDDs (Fig. 22) [252, 253]. Table 4 describes the common imaging features of miscellaneous NDDs.

**Table 4:** Synopsis of imaging features of miscellaneous neurodegenerative disorders

	PsD	CJD	HD	ALS	CTE	CAA
sMRI		Cortical ribboning and basal ganglia and thalamic (hockey stick) hyperintensity of T2 or DWI	Atrophy of striatum with ex vacuo dilated “box-shaped” frontal horns of the lateral ventricles	T2 hyperintensity of CSTs and perirolandic regions	Increased preponderance of cavum septum pellucidum with generalized cerebral atrophy	Inflammatory type: lobar vasogenic edema with leptomeningeal enhancement Amyloidoma—solitary enhancing mass with surrounding edema
SW/T2*				Increased iron deposition in motor cortex		Lobar and subcortical microhemorrhages and superficial siderosis
MRS		Decreased NAA of affected regions		Decreased NAA in the CSTs		
DTI			Increased FA and MD in the basal ganglia	Reduced FA in the CSTs	Reduced FA in superior and inferior longitudinal fasciculus, corona radiate, cerebral peduncle, uncinate fasciculus, anterior thalamic radiations, and striatum	
rsfMRI				Hypoconnectivity of the motor network		
ASL						
FDG-PET	Generally normal	Asymmetric hypometabolism in the frontal and parietal cortices with or without decreased activity in the in the basal ganglia	Hypometabolism in the striatum and frontal and temporal lobes	Hypometabolism of the motor/perirolandic and frontal cortices and occipital lobes	Hypometabolism in the frontotemporal lobes	Hypometabolism in pattern similar to AD with increasing occipital to global or PCC ratio
Amyloid PET	Generally normal	No significant uptake			Variable uptake	Uptake in pattern similar to AD with increasing occipital to global or PCC ratio
Tau PET	Generally normal	Off-target binding of certain tracers felt to be related to astrocytosis		[11C]PBB3 uptake in the CSTs	Uptake in the frontotemporal lobes including the medial temporal lobes	Neocortical uptake correlates with worsening cognition
DAT/Dopamine imaging			Decreased striatal dopamine receptor binding			

*sMRI* structural magnetic resonance imaging, *MRS* Magnetic resonance spectroscopy, *DTI* Diffusion tensor imaging, *rsfMRI* Resting-state functional magnetic resonance imaging, *ASL* Arterial spin labeling, *FDG* Fluorodeoxyglucose, *PET* Positron emission tomography, *DAT* Dopamine transporter, *CAA* Cerebral amyloid angiopathy, *AD* Alzheimer’s disease, *PCC* Posterior cingulate cortex, *CTE* Chronic traumatic encephalopathy, *FA* Fractional anisotropy, *ALS* Amyotrophic lateral sclerosis, *CSTs* Corticospinal tracts, *NAA* N-acetylaspartate, *HD* Huntington’s disease, *CJD* Creutzfeldt–Jakob disease, *DWI* Diffusion-weighted imaging, *PsD* Pseudodementia

## Conclusion

A wide variety of advanced multiparametric MRI and molecular imaging techniques are now available to increase diagnostic confidence of neurodegenerative syndromes, with ongoing research to thrust more of these techniques into a wider clinical role. Combined PET/MRI is an attractive imaging modality to provide a comprehensive workup of NDDs in a single imaging session. Ultimately, these techniques may be of use in selecting for and following up patients on monoclonal antibody therapy.

## Data Availability

Not applicable.

## Abbreviations

11C-DED: 11C-deuterium-l-deprenyl
3R: Three repeat
4R: Four repeat
AD: Alzheimer’s disease
ARIAs: Amyloid-related imaging abnormalities
ASL: Arterial spin labeling
Aβ: Amyloid-beta
C-11: Carbon-11
C9ORF72: Chromosome 9 open reading frame 72
CAA: Cerebral amyloid angiopathy
CBD: Corticobasal degeneration
CBS: Corticobasal syndrome
CJD: Creutzfeldt–Jakob disease
CMRgluc: Cerebral metabolic rate of glucose
CTE: Chronic traumatic encephalopathy
CTSs: Corticospinal tracts
DAT: Dopamine transporter
DLB: Dementia with Lewy bodies
DMN: Default mode network
DTI: Diffusion tensor imaging
DWI: Diffusion-weighted imaging
F-18: Fluorine-18
FA: Fractional anisotropy
FDG-PET: 2-[18F]fluoro-2-deoxy-d-glucose positron emission tomography
FTD: Frontotemporal dementia
FTLD: Frontotemporal lobar degeneration
FUS: Fused-in-sarcoma
I-123-FP-CIT, DaTSCAN: Iodine-123 N-ω-fluoropropyl-2β-carbomethoxy-3β-[4-iodophenyl] nortropane
LC: Locus coeruleus
lvPPA: Logopenic variant primary progressive aphasia
MAO: Monoamine oxidase
MAPT: Microtubule-associated protein tau
MCI: Mild cognitive impairment
MD: Mean diffusivity
MMSE: Mini-mental state examination
MoCA: Montreal cognitive assessment
MRE: Magnetic resonance elastography
MRI: Magnetic resonance imaging
MSA: Multisystem atrophy
MSA-C: Multisystem atrophy with dominant cerebellar dysfunction
MSA-P: Multisystem atrophy with parkinsonian features
MTI: Magnetization transfer imaging
MTR: Magnetization transfer ratio
NAA: N-acetylaspartate
NDD: Neurodegenerative diseases
nfPPA: Nonfluent/agrammatic primary progressive aphasia
NFTs: Neurofibrillary tangles
PCA: Posterior cortical atrophy
PCC: Posterior cingulate cortex
PD: Parkinson’s disease
PDD: PD-related dementia
PiB: Pittsburgh compound B
PSEN1: Presenilin 1
PSEN2: Presenilin 2
PSP: Progressive supranuclear palsy
ROI: Region of interest
rsfMRI: Resting-state functional MRI
SPECT: Single-photon emission computed tomography
ss-SPM: Single-subject statistical parametric mapping
SUVgluc: Standard uptake value of glucose
SUVr: Standardized uptake value ratio
svPPA: Semantic primary progressive aphasia
TDP-43: TAR DNA-binding protein 43
VaD: Vascular dementia
WMHs: White matter abnormalities/hyperintensities
